# Hydrodynamic response of an Antarctic glacial bay to cross-bay winds and its potential impact on primary production

**DOI:** 10.1038/s41598-025-34031-1

**Published:** 2026-01-30

**Authors:** Maria Osińska, Agnieszka Herman

**Affiliations:** 1 University of Gdańsk, Jana Bażyńskiego 8, 80-309 Gdańsk, Poland; 2 Institute of Oceanology of Polish Academy of Sciences, Powstańców Warszawy 55, 81-712 Sopot, Poland

**Keywords:** Admiralty Bay, Southern Ocean, Numerical modelling, Physical oceanography, West Antarctic Peninsula, GMW, Climate sciences, Ecology, Ecology, Ocean sciences

## Abstract

Antarctic glacial bays are important, productive regions of the Southern Ocean. Certain glacial bays, including our research area, Admiralty Bay, are less favorable for phytoplankton growth due to wind-enhanced high energy levels, but they still host localized biological blooms. Westerly winds are predominant in Admiralty Bay; the strongest storms are from the east. These winds act perpendicular to the main axis of the bay. This study investigates the impact of cross-bay winds on the bay’s hydrodynamics and its potential effects on primary production. A hydrodynamic model, coupled with a Lagrangian model tracking potential iron sources, was run under seven wind scenarios. Results indicate that all winds reduce water column stratification, but energy increase rates and circulation pattern shifts vary with wind direction. Westerly winds restrict outflow and promote the formation of submesoscale eddies near inner inlet openings, concentrating water masses that are expected to be iron-rich, potentially stimulating phytoplankton growth. Conversely, easterly winds enhance outflow, flushing bay waters and likely negatively impacting productivity. Limited observational and satellite-derived biological data provide supportive evidence for the model-based hypothesis that the direction of cross-bay winds, rather than just their magnitude, significantly influences local productivity.

## Introduction

Due to their relatively high levels of primary production, Antarctic glacial bays of the West Antarctic Peninsula (WAP) region are essential for the Southern Ocean (SO) ecosystem. Phytoplankton blooms are prevalent in these areas, boosting the numbers of Antarctic krill, an important fish, bird, and marine mammal food source^1^. The high productivity observed in glacial bays can be attributed to the availability of iron, which is a limiting factor for primary production in the SO^2^. Iron is supplied to the SO through various mechanisms, the most significant for coastal waters being sediment resuspension through upwelling, sea-ice melt, dust deposition via precipitation, and glacial runoff^3–6^.

The presence of iron sources alone is insufficient to establish a biological bloom, as many glacial bays are highly dynamic and are characterized by significant water exchange between the bay and the ocean, which flushes out nutrient-rich waters^7,8^. Therefore, in these bays, favorable hydrodynamic and geomorphological conditions are necessary for phytoplankton growth. Strong water column stratification has been found to be important for high levels of primary production^9^. If the nutrient source are the iron-rich bottom waters (BW), a mechanism must exist that elevates it to the euphotic layer. In an energetic environment, accumulation zones of nutrient-rich waters are required to give time for the phytoplankton bloom to emerge and for higher-level consumers to utilize it. Accumulation zones can develop when vorticity increases and eddies form, often shaped by the complexities of the seabed, such as mounts and basins^10,11^. Such submesoscale eddies are known to facilitate the development of localized hotspots of primary production^12^.

Water column stratification, upwelling, elevated vorticity, and subsequent formation of water accumulation areas are influenced by the winds^9,10,13^. The impact of downfjord and upfjord winds on glacial bay circulation is well established, supported by numerous studies that investigated their effects on the hydrodynamics of elongated fjords where these winds prevail^13,14^. The Antarctic Peninsula coastline is highly intricate, comprising more than 100 bays adjacent to over 800 glaciers^15^. Numerous WAP bays are broad, allowing winds to affect them in the cross-bay axis; however, the impact of these winds on the bay hydrodynamics and its implications for the local environment remains unknown.

**Fig. 1:**
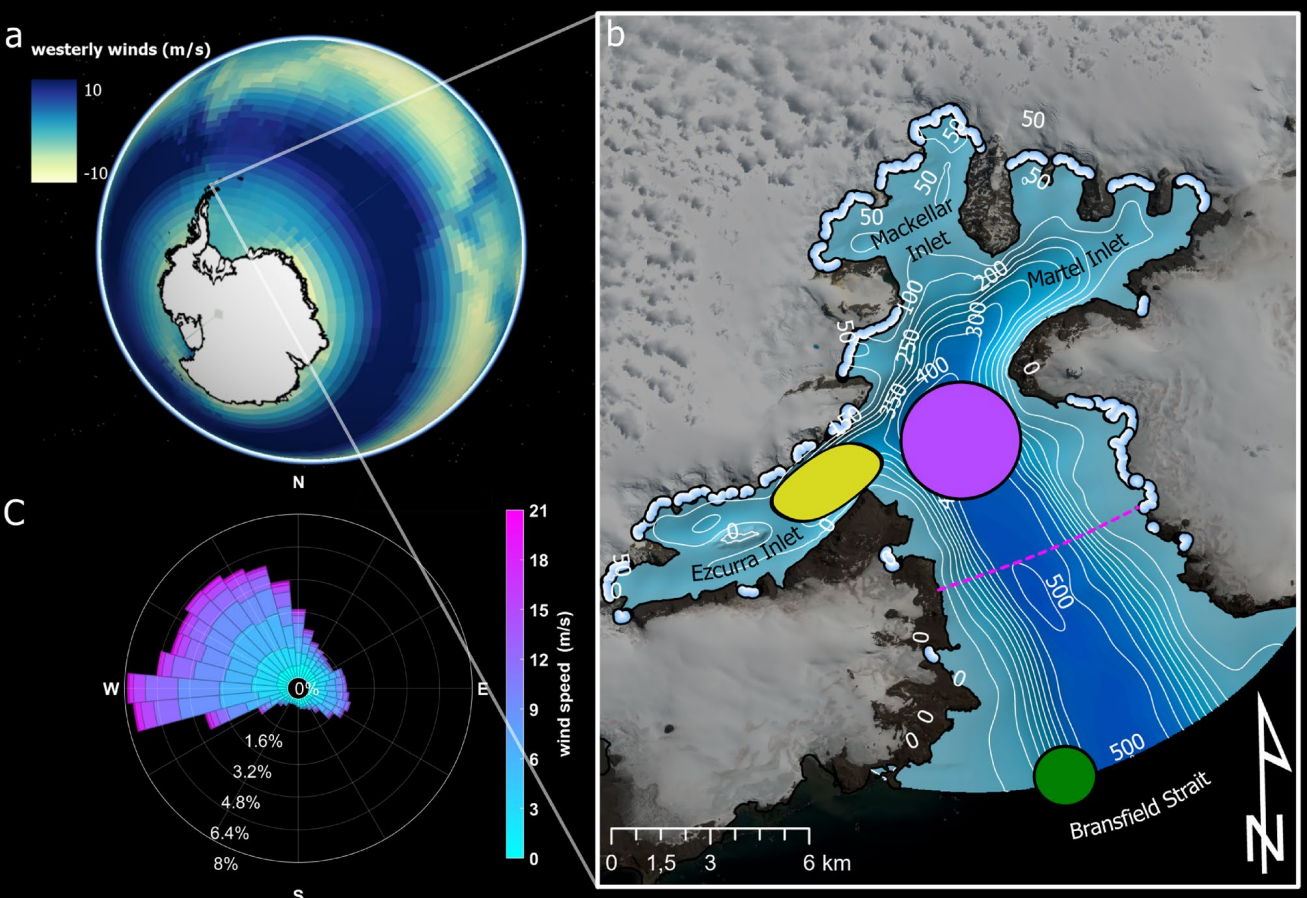
Admiralty Bay winds and bathymetry, (**a**) westerly wind belt around Antarctica, the colormap shows the west wind component of long-term mean winds from 1991 to 2020^18^, (**b**) bathymetric map of Admiralty Bay^7^; thick light blue lines indicate glacial/water boundaries^19^ and starting points of GMW particle tracking; purple circle–starting area of BW particle tracking; green circle–starting area of OB particle tracking; pink dashed line–boundary of inner model domain; yellow field–known feeding hotspot; background–Sentinel imagery 29.12.2021, (**c**) wind rose for Admiralty Bay from 15.12.2018 to 1.03.2023^20^.

Admiralty Bay (AB), our research area, is located in the southwest of King George Island (KGI), South Shetland Islands, approximately 120 km northwest of the Antarctic Peninsula (Fig. 1a). The main body of AB, measuring 18 km in length and 8 km in width, is characterized by weak water column stratification and a low Rossby radius of deformation (*A*), which averages 1.3 km throughout the year (estimate based on *in situ* measurements^7^). This classifies it as a ’broad bay’ where rotational dynamics play a significant role. Due to the absence of a pronounced sill (Fig. 1b) AB’s circulation is primarily driven by oceanic forcing, leading to a vigorous exchange of water between the ocean and the bay^7^.

AB is known for its large and constant presence of penguins and marine mammals and, in fact, was named by British whalers and sealers who have been hunting them here since the 19th century^16^. Due to its rich animal populations, it is now designated both as an Antarctic Specially Managed Area and partially as an Antarctic Specially Protected Area. Primary production in AB is lower than in some other glacial bays due, among other factors, to its high dynamics stimulated by strong winds^8,17^. Despite this, the area has experienced significant temporary phytoplankton blooms^8,17^. Also, repeated localized feeding hotspots of whales and penguins have been observed by the staff of Arctowski Polish Antarctic Station in an area marked in yellow in Fig. 1b (an example of such a feeding frenzy can be seen in Supplementary Video 1 online).

The bathymetry of AB, characteristic of bays in WAP, exhibits a deep fault exceeding 500 m at its center, with shallower inlets extending from the main body of the bay in various directions (Fig. 1b). Twenty marine-terminating glaciers, seen in Fig. 1b, are located along the AB coastline, releasing freshwater into the bay, primarily during austral summer and early autumn^7^. This freshwater mixes with the surrounding seawater, creating glacially modified waters (GMW). Previous research in AB confirms that GMW is a significant source of iron. Iron concentrations in sediments ranged from 2.40 $\times$ $10^2$ to 5.15 $\times$ $10^3$ $\upmu$g/L measured in highly turbid glacial plume waters in close proximity to the ice front^21^. Dissolved iron concentrations in surface waters ranged from 8.49 x $10^{-4}$ to 1.74 x $10^{-3}$ $\upmu$g/L, with peak levels observed closest to the glaciers^22^. These concentrations exceed typical levels found in the SO (previous studies have reported particulate iron concentrations in the coastal WAP and SO Atlantic sector ranging from 1.01 x $10^{-5}$ to 7.99 x $10^{-3}$ $\upmu$g/L, and dissolved iron concentrations ranging from 5.58 x $10^{-6}$ to 4.41 x $10^{-4}$ $\upmu$g/L^6,23^). Furthermore, although based on limited observations, Brandini and Rebello^24^ demonstrated a positive relationship between upwelling events and chlorophyll-a (*Chl-a*) concentrations in the AB, suggesting that upwelled BW also acts as a nutrient source for local phytoplankton communities. Based on this earlier research in AB and the recent findings by Annet et al.^4^, as well as considering the region’s low precipitation levels^25^ and the decreasing sea ice extent^26^, it seems reasonable to assume that BW and GMW are the primary sources of iron in AB.

Westerly and northwesterly winds are the prevailing wind directions in AB (Fig. 1c). In recent years, due to the climate-change-related strengthening of circumpolar westerly winds (Fig. 1a), AB has experienced an increase in the western wind component^27,28^. However, the analysis of data from 60 years of *in situ* observations has demonstrated that the 100 strongest wind events in this region were primarily caused by easterly and southeasterly winds^27^. Hence, the most frequent winds (westerly) and the strongest winds (easterly) are impacting AB waters perpendicularly to the main axis of the bay.

In short, AB is a broad glacial bay characterized by a weak water column stratification and an energetic water exchange with the open ocean. The capacity of this seemingly dynamic environment to sustain a vibrant ecosystem, as evidenced by frequently observed feeding hotspots, presents a conundrum that this study aims to address. We hypothesize that the apparent paradox can be explained by the additional forcing mechanism introduced to the system by cross-bay winds, which stimulate localized areas of biological productivity. Therefore, this study firstly seeks to understand the impact of cross-bay winds on the hydrodynamics of AB; further, it aims to explore the potential influence of these wind patterns on the development of localized biological blooms. Studying such processes requires a comprehensive approach combining observations and modelling. Empirical investigations in WAP glacial bays are inherently challenging. While extensive *in situ* datasets, such as that compiled by Osińska et al.^29^, provide valuable insights into the variability of water properties, the logistical difficulties and safety concerns associated with conducting measurements, particularly under strong wind conditions, limit their scope. Furthermore, persistent cloud cover significantly restricts the utility of satellite data in visible bands (see Methods). Therefore, to isolate and examine the specific impact of cross-bay winds, we employ a high-resolution hydrodynamic model coupled with Lagrangian particle tracking model. This approach allows us to systematically analyze the effects of varying wind direction and magnitude on the bay’s circulation, stratification, and particle transport. Finally, available *in situ* biological and satellite data are analyzed to assess if these wind-driven hydrodynamic changes in fact influence primary production levels in AB.

## Results

### Cross-bay wind effects on kinetic energy, stratification, and transport

**Fig. 2:**
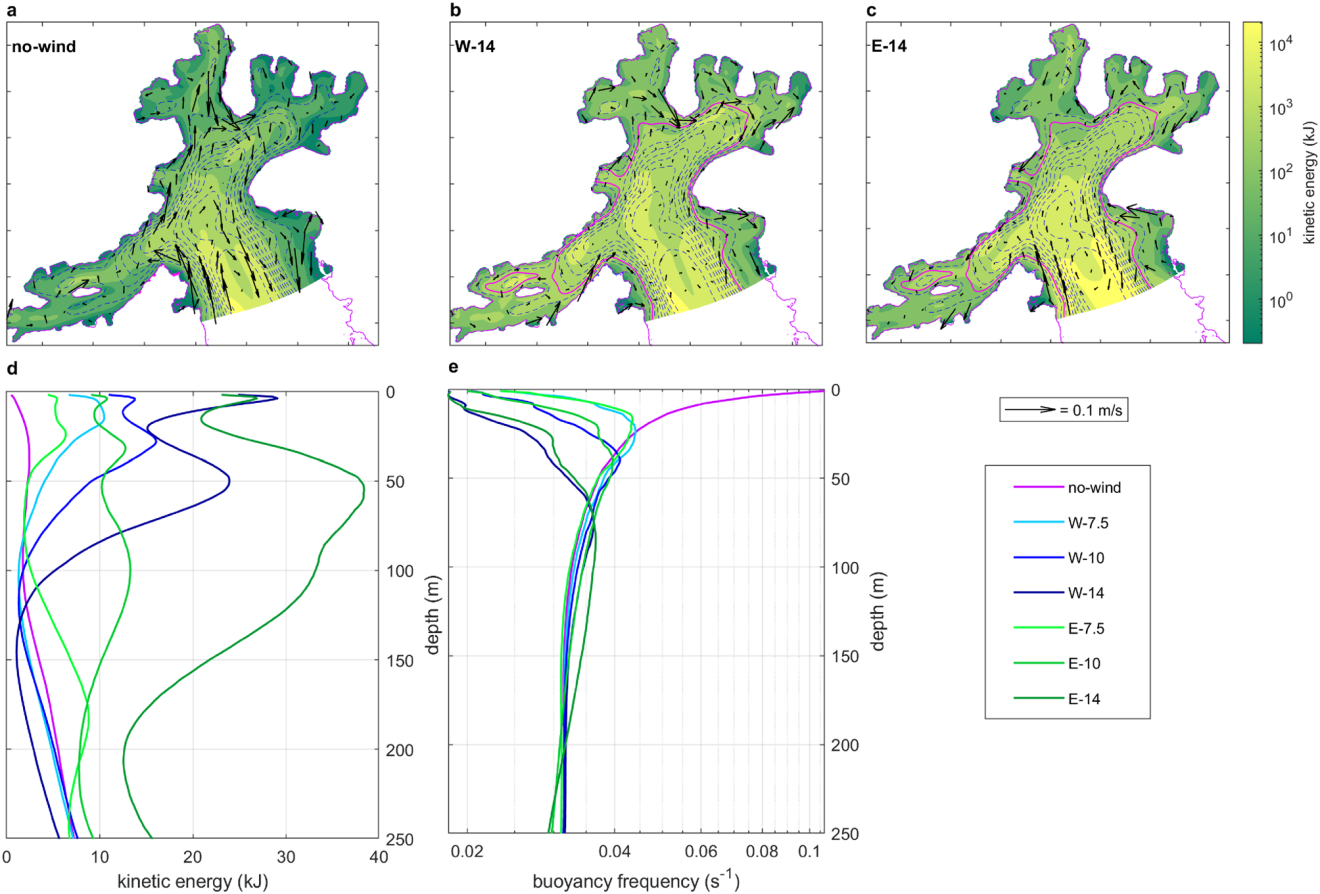
Shifts in AB hydrodynamics caused by wind forcing, (**a–c**) spatial variations in *no-wind*, *W-14*, and *E-14* scenarios; colors represent kinetic energy integrated across the water column; arrows indicate depth-averaged horizontal velocity vectors; blue dashed lines show isobaths; magenta line shows the isobath equal to $D_E$, (**d**) vertical profiles of the spatially mean kinetic energy, (**e**) vertical profiles of the spatially mean buoyancy frequency (*N*). Note: all plots show mean values from Dec 7, 2021, to Jan 9, 2022 (33 days).

Osińska and Herman^7^ characterized the general circulation pattern within AB, revealing a system of two cyclonic cells that regulate water exchange between the Bransfield Strait and the bay, as depicted in Fig. 2a. Ocean waters enter the bay primarily through a strong inflow current along its western boundary, while GMW is exported in the surface layer via a southerly outflow current in the east. To investigate the influence of cross-bay winds on this circulation pattern, the AB hydrodynamic model^7^ was run in seven scenarios: a reference case without wind forcing (*no-wind* scenario) and three scenarios of increasing westerly and easterly winds each (Table 1). The used wind speeds of 7.5, 10, and 14 m/s correspond to the 50th, 75th, and 90th percentiles of all wind magnitudes recorded in AB.

**Table 1:** Summary of the model runs.

	No-wind	W-7.5	W-10	W-14	E-7.5	E-10	E-14
Wind direction (deg)	-	270	270	270	90	90	90
Wind magnitude (m/s)	0	7.5	10	14	7.5	10	14
Estimated Ekman layer depth $D_E$ (m)	0	33	50	77	33	50	77

Figure 2a–c presents a comparison of kinetic energy content and depth-averaged horizontal velocities in *no-wind*, strong westerly wind (*W-14*), and strong easterly wind (*E-14*) scenarios (for maps in higher resolution for all scenarios see Supplementary Figs. S1–S7 online). The vertical profiles of the mean kinetic energy from all scenarios are given in Fig. 2d. The results indicate that energy levels in AB waters increase with increased wind forcing; however, depending on the wind direction, distinct horizontal and vertical patterns are observed. Under westerly wind conditions (Fig. 2b) energy increases in both the main body of the bay (in the whole domain increase of 25% in *W-7.5*, 61% in *W-10*, and 107% in *W-14* relative to the *no-wind* scenario) and most significantly in the inlets perpendicular to the bay’s main axis: Ezcurra and Martel Inlets (EI and MI; Fig. 1b). Since these inlets are shallower than the main body of AB, the increase in energy in spatially averaged vertical energy profiles is mostly seen within the upper 100 m (Fig. 2d). Easterly winds (Fig. 2c) result in a greater increase in energy (*E-7.5* 30% , *E-10* 127% , and *E-14* 352% increase). In contrast to westerly wind scenarios, this rise concentrates predominantly within the main body of AB, subsequently enhancing the velocities of the cyclonic circulation cell (for example, the inflow current in *E-14* is on average four times stronger than in the *no-wind* scenario). The rise in energy due to strengthening easterly winds is observed throughout the entire water column (Fig. 2d).

Since the intensification of easterly winds enhances circulation within the main body of AB, it leads to a significant increase in outflow from the model domain, with outflow of waters increasing by 38–108% as easterly winds strengthen. The volume of water exported from the AB boundary increases by only 3–15% with the strengthening of westerly winds (see Supplementary Fig. S8 online).

The stratification of the water column has been assessed through the buoyancy frequency (*N*)^30^ (Methods). AB waters are well mixed even in the absence of wind forcing (Fig. 2e). A maximum *N* of 0.11 s$^{-1}$ is recorded in the upper layers in the *no-wind* scenario due to the presence of fresh GMW in the surface layer. *N* decreases with increasing wind magnitude regardless of wind direction. Low *N* corresponds to reduced *A* values: averaging 1.16 km in the absence of wind and approximately 0.6 km under all wind-induced scenarios, consistent with aforementioned *A* estimates derived from observations^7^.

### Particle tracks variability

**Fig. 3:**
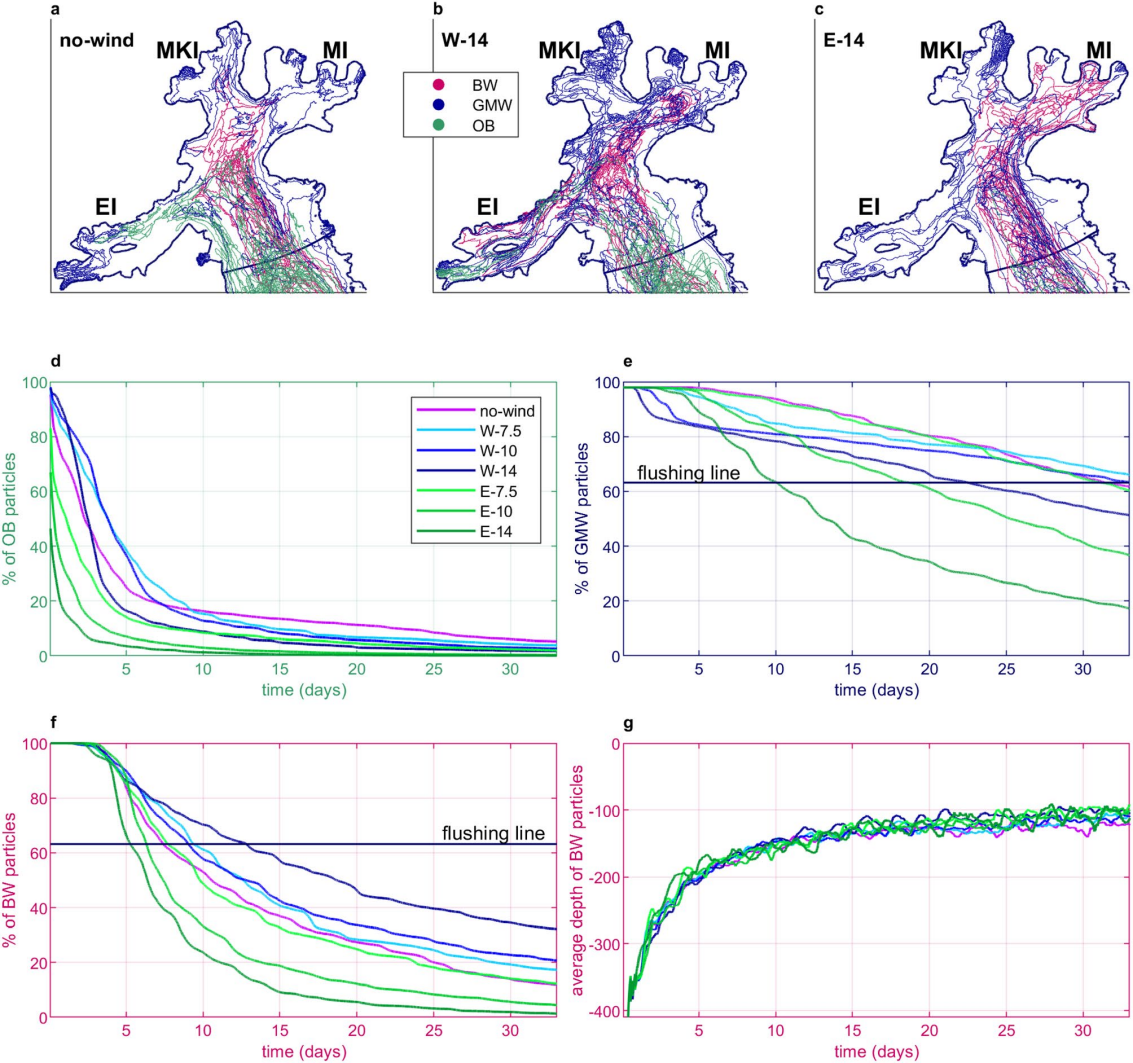
Pathways and residence time of tracked water particles in different wind scenarios, (**a–c**) selected trajectories of BW, GMW, and OB particles in *no-wind*, *W-14*, and *E-14* wind conditions, respectively, (**d**) residence time of OB particles in the model domain, (**e**) residence time of GMW particles, (**f**) residence time of BW particles, (**g**) average depth of BW particles over time.

To further investigate the water mass transport within AB, a Lagrangian particle tracking model was coupled to the hydrodynamic model. Three types of water masses were monitored: two expected sources of iron, GMW and BW, as well as open boundary waters (OB). OB refers to waters entering the AB from the Bransfield Strait, with their paths illustrating the influence of local winds on the penetration of ocean waters into the bay. In Fig. 1b, the starting areas of these three particle groups are shown. Figure 3a–c illustrates 25 randomly selected routes for each group under *no-wind*, *W-14*, and *E-14* wind conditions.

The statistics of OB particle tracks (green pathways in Fig. 3a–c and their residence time across model scenarios shown in Fig. 3d) indicate that easterly winds limit the oceanic influx into AB–in the *E-14* scenario: only 50% of the initially introduced particles remain in the domain for more than one day (the average residence time varies from 1.40 to 0.73 days with different magnitudes of easterly winds). This is illustrated in Fig. 3c, where OB routes are nearly absent. In contrast, westerly winds have a comparable impact on the ocean penetration of AB waters as with no winds, with the average OB particle residence time in westerly wind scenarios varying from 3.75 to 5.69 days compared to 5.46 days in a *no-wind* scenario.

GMW (dark blue pathways in Fig. 3a–c; residence time in Fig. 3e) is exported from AB most rapidly during strong easterly winds. To assess this effect, we calculate the flushing time, which is defined as the duration needed to reduce the initial number of particles by a fraction of $1-\exp ^{-1}\approx 0.63$^31^. In *E-14*, GMW particles are flushed from AB within 10 days, which is twice as fast as in the *E-10* and *W-14*, and three times faster than in all other scenarios.

The residence time of BW particles in AB is also correlated with wind direction and magnitude (dark pink pathways in Fig. 3a–c; residence time in Fig. 3f). The stronger the westerly winds, the longer the BW particles stay in AB. The opposite effect arises from easterly winds. The BW particle flushing time between the *E-14* and *W-14* scenarios varies by 8 days (5 and 13 days, respectively). In *E-14,* after 33 days of modeling, less than 2% of BW particles remain in the AB, while in *W-14,* one-third remain inside. With westerly winds, similarly to GMW, BW pathways extend further into smaller AB inlets (Fig. 3a–c). Across all simulated scenarios, BW particles were observed to rise from the seabed to an average depth of 100 m within approximately 15 days (Fig. 3g). This indicates that, regardless of wind conditions, the interaction between oceanic forcing and the shallowing bathymetry of AB consistently generates upwelling, effectively lifting BW into the higher ocean strata.

### Formation of GMW and BW accumulation areas

**Fig. 4:**
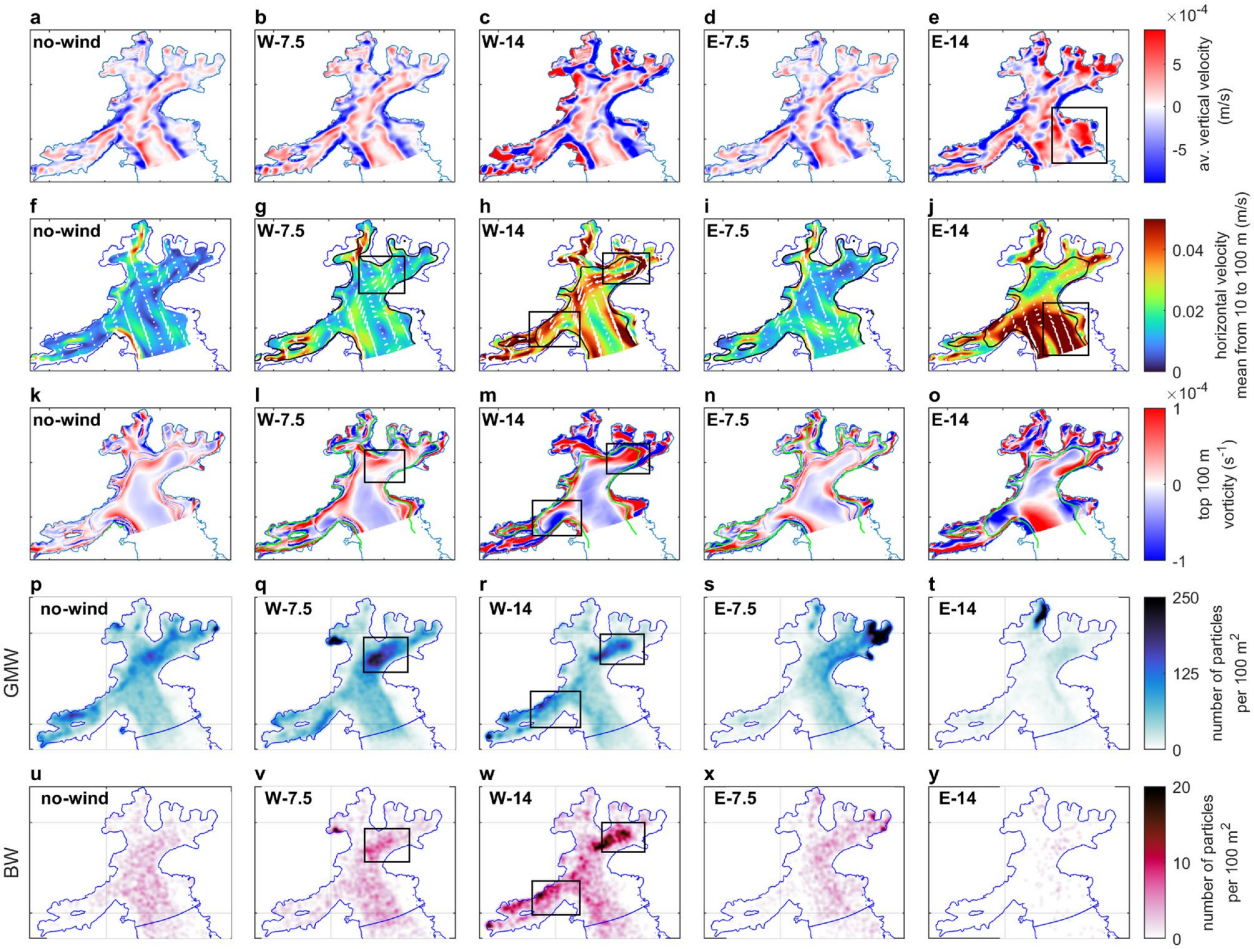
Changes in AB flow pattern in five wind scenarios, (**a–e**) vertical velocities > |0.05| cm/s; positive values indicate movement upwards, (**b–e**) anomalies in relation to *no-wind* scenario values, (**f–j**) horizontal velocities averaged across 10–100 m depth; black line – the isobath corresponding to $D_E$, (**k–o**) vorticity averaged across 10–100 m, with bathymetry; green line – $D_E$, (**p–t**) GMW density maps after 33 days from release, (**u–y**) BW density maps after 33 days from release. *Note*: Fig. 4 a-o. show mean values from Dec 7, 2021, to Jan 9, 2022 (33 days).

To gain a more precise understanding of the factors that contribute to the observed disparities in residence time of GMW and BW particles in the modeled scenarios, it is necessary to examine vertical and horizontal flow pattern shifts caused by cross-bay winds (Fig. 4).

During *no-wind* conditions, the main body of AB experiences downwelling along its eastern and western margins and upwelling in the center. This upwelling, seen in all scenarios, causes the aforementioned continuous lifting of BW particles. This pattern is enhanced by the increasing wind forcing seen in *W-7.5*, *W-14*, and *E-7.5* scenario results (Fig. 4a–d; note that the values in panels b–e are anomalies with respect to those in panel a). A strong easterly wind of 14 m/s (*E-14*) generates a notable pressure gradient from east to west (difference of approximately 0.25 dbar between the west and east sides of AB), which induces upwelling along the eastern wall of the main body of AB (area indicated in the box in Fig. 4e). This upwelling strengthens the easterly outflowing current (see the box in Fig. 4j). The Ekman transport under easterly winds is directed southward toward the bay’s mouth, thereby further increasing the outflowing current (Fig. 4j). The enhancement of the outflow initiated by the upwelling explains why *E-14* exhibits qualitatively different behavior compared to other scenarios. This scenario is associated with the highest energy levels, the highest transport volumes out of the bay, and thus the fastest export of OB, GMW, and BW particles from the model domain (Figs. 2d and 3).

The low *A* values (0.6 to 1.6 km in all scenarios) relative to the bay width ($\sim$ 8 km) result in a cyclonic circulation in AB, with the strongest currents flowing along the bay’s coastline (Fig. 2a). The shallower the waters, the more these currents can be influenced by the additional curl generated by the wind. Therefore, the larger the ratio of the Ekman layer depth ($D_E$) to the total water depth, the larger the increase in vorticity (see vorticity relation to $D_E$ in Fig. 4k–o, green lines show isobaths equal to $D_E$).

Despite the general cyclonic circulation in AB, an opposite, anticyclonic circulation cell exists in the surface waters of the main body of AB (Fig. 4f). As the westerly winds increase, there is a rise in horizontal flow velocities, more significant within the inner waters of MI and EI compared to easterly wind scenarios (Fig. 4f–j, and in high resolution in Supplementary Figs. S9–S15 online). At the inner inlets’ openings, eddies are formed: in the *W-7.5* scenario, a cyclonic eddy forms between the Mackellar Inlet (MKI) and MI openings, while in the *W-14* scenario, much stronger but elongated eddies appear at the mouths of MI (cyclonic) and EI (anticyclonic; see the boxes in Fig. 4g–h and l–m; vorticity for all scenarios is shown in Supplementary Figs. S16–S22 online). These eddies form when outflowing currents from MI and EI, moving along their southern edge, are affected by the wind curl, which redirects their flow northward, closing the circulation cells. Additionally, in the main body of AB, northerly Ekman transport enhances the surface anticyclonic cell, which reduces the net outflow from the bay (Fig. 4h). This explains why the westerly wind speed does not increase the volume of transport out of the bay, even while the system’s energy levels rise.

The entrapment of particles occurs in nonlinear eddies where rotational velocities exceed those of the surrounding fluid^32^. In *W-75*, *W-10*, and *W-14*, the horizontal velocities in the vortices at the mouths of EI and MI exceed those of the surrounding waters, and there is no significant increase in vertical mixing, providing favorable conditions for accumulation (Fig. 4b, c, g, h, *W-10* in Supplementary Fig. S11 online). The eddies near the mouth of EI, MI, and within the large circulation cell in the main AB interchange waters, and trap particles between them for extended periods of time. Therefore, the eddies at the mouths of the inner inlets become accumulation areas of GMW and BW particles. This is confirmed by the density maps of GMW and BW particles after 33 days of modeling (boxes in Fig. 4q, r, v, and w, corresponding to the same locations on the horizontal flow maps in panels g and h, and increased vorticity in panels l and m).

In the *no-wind* scenario, a surface eddy is formed at the same location as in the *W-7.5* scenario, corresponding to an area with increased amounts of GMW and BW particles. However, due to its lower velocity, its entrapment effect is weaker (Fig. 4f, k, p and u). As seen previously through residence-time analysis, the presence of easterly winds leads to a reduction in the amounts of GMW and BW particles within AB. Nonetheless, accumulation is observed in the *E-7.5* scenario in the east of MI (Fig. 4s and x). Strong easterly winds (*E-14*) create an anticyclonic vortex at the entrance to EI. However, it is not categorized as a nonlinear eddy because its velocity is lower than that of the adjacent strong outflow current from the bay; thus, the waters within it are not accumulated but are rapidly flushed out (Fig. 4j, o, t and y).

## Discussion

**Fig. 5:**
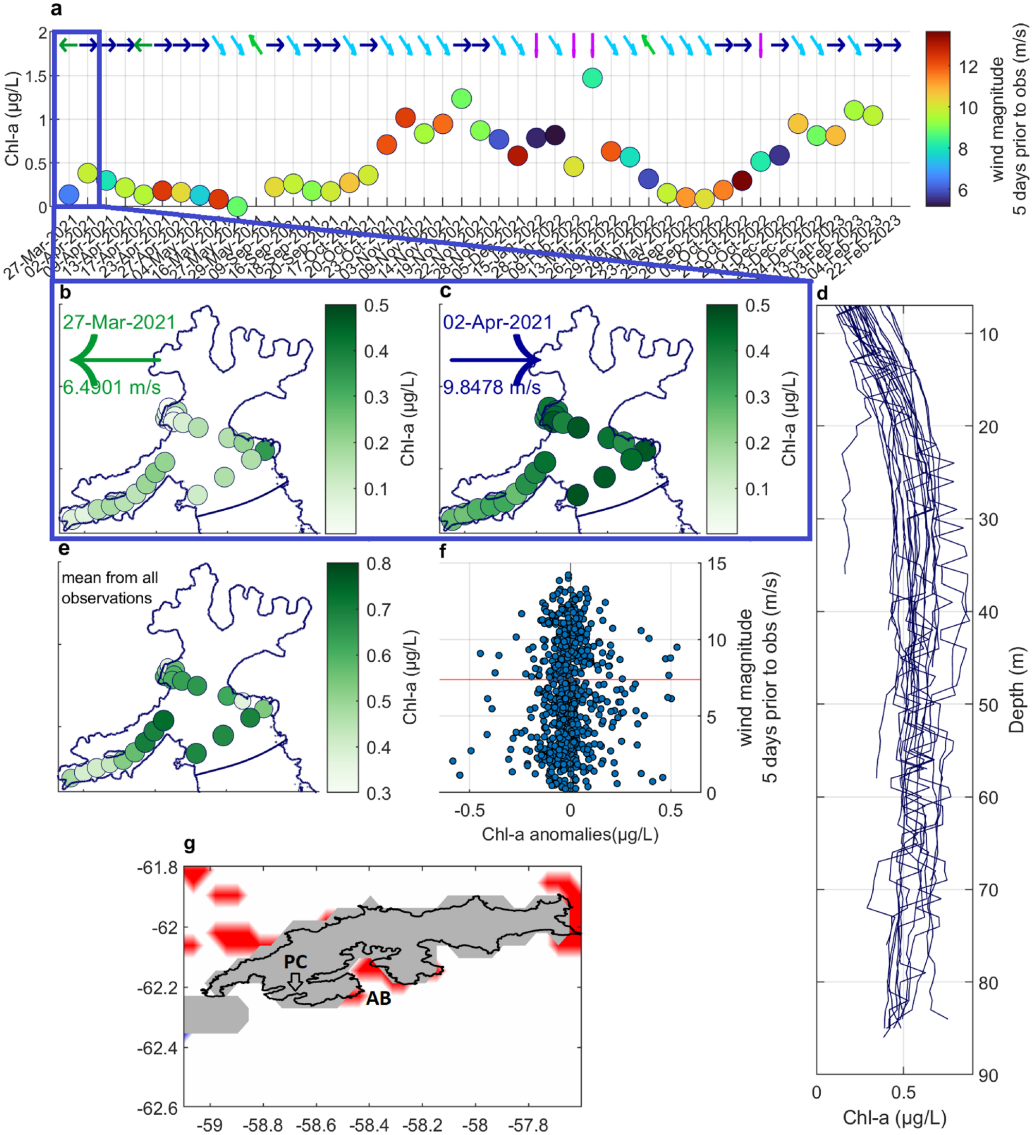
*Chl-a* content dependent on wind conditions; measured and from GlobColour, (**a**) daily measured mean *Chl-a* values relationship with mean wind magnitude and predominant wind direction in 5 days prior to the measurement; the blue box highlights two days shown in detail in panels (**b**) and (**c**), (**b**) depth-averaged *Chl-a* values measured on 27th of Mar 2021, (**c**) depth-averaged *Chl-a* values measured on 2nd of Apr 2021, (**d**) *Chl-a* values from all measurements at each sampling site as function of depth, (**e**) mean *Chl-A* from all measurements and depth-averaged at each site, (**f**) mean daily GlobColour *Chl-a* anomalies relationship with wind magnitudes 5 days prior to the observation; red line – 7.5 m/s wind magnitude, (**g**) areas around KGI in which U-test revealed a significant disparity between GlobColour *Chl-a* anomalies after westerly/easterly wind forcing; red areas – a significantly higher *Chl-a* values after westerly winds; white areas – no significant relation; grey areas – no data.

In both AB and Potter Cove (PC; a small but well-studied embayment of KGI, approximately 15 km west of AB, marked in Fig. 5g), significant phytoplankton blooms were observed in 2010 and 2017, with *Chl-a* values exceeding 15 $\upmu$g/L. These blooms have been attributed to particularly calm conditions that stabilized the water column, promoting phytoplankton presence in the euphotic zone^8,9,17^. Although vast blooms like those of 2010 and 2017, when they appear, are important for the local ecosystem, their occurrence frequency and thus significance is limited. Calm wind periods are rare in AB and throughout the WAP, and climate change is likely to further limit them^28^ (Fig. 1c). Therefore, it is crucial to understand how phytoplankton growth occurs under more typical, yet less favorable conditions.

We postulate that in these circumstances nonlinear submesoscale eddies play a significant role. This is supported by previous findings showing that both cyclonic and anticyclonic eddies can stimulate phytoplankton blooms by concentrating nutrient-rich particles^12^, reducing vertical mixing and increasing light availability in the surface layers^33^. Our modeling results indicate that westerly winds are favorable for the formation of submesoscale eddies in AB, leading to the accumulation of BW and GMW particles. Consequently, we suggest that these eddies can serve as the foundation for biological hotspots, in which the concentration of iron-rich waters (BW and/or GMW) in the euphotic layer fuels phytoplankton growth. Crucially, our modeling indicates that this process is explicitly associated with westerly wind conditions, while easterly winds induce the opposite effect.

To investigate whether cross-bay winds indeed significantly influence phytoplankton growth in AB, we analyzed the relationship between *Chl-a* variability and concurrent wind conditions. While *Chl-a* reflects pigment levels rather than photosynthetic rates or biomass, its photosynthetic role makes it a proxy for primary production^34^. We used *Chl-a* data from Osińska et al.^29^ (Fig. 5b, c, e for locations; Fig. 5a for dates) and the satellite-derived Copernicus GlobColour product^35^ (GlobColour; Methods). Both datasets have limitations: the observational data lack records from high wind and easterly/southeasterly wind events and do not cover some key areas like the MI mouth. The GlobColour product has a coarse spatial resolution (4 km) and a 45.80% uncertainty. Nevertheless, Osińska et al.’s measurements are among the most comprehensive datasets that describe water property variability in Antarctic glacial bays, and GlobColour provides the only high-temporal-resolution *Chl-a* estimates for this region. Therefore, although far from perfect, these datasets provide the most reliable *Chl-a* information available.

The variability of *Chl-a* levels from these two data sources, in relation to wind conditions, is illustrated in Fig. 5. Compared to other glacial bays in WAP, AB exhibits a comparatively low *Chl-a* values, with daily mean values rarely exceeding 1 $\upmu$g/L, and maximum values recorded at approximately 40 m depth (Fig. 5a and d; Supplementary Fig. S23 online for GlobColour data)^17,24,36,37^. Previous research attributed the reduced primary production in AB and PC to the strong wind-induced mixing and to the weak stratification in these bays^8,9^. If this hypothesis were true, a negative correlation would be expected between wind speed and *Chl-a* anomalies. However, we found no statistically significant correlation between the GlobColour *Chl-a* anomalies and wind speed (correlation coefficient $r=-0.10$; Fig. 5f). Furthermore, several instances of positive *Chl-a* anomalies were observed after periods of strong winds, indicating that phytoplankton can reach relatively high concentrations even after wind-driven reduction of water-column stratification (Fig. 5a and f; note the line in Fig. 5f at 7.5 m/s wind magnitude, above which reduced *N* values are observed, as shown in Fig. 2e). These observations suggest that strong vertical mixing is not a prerequisite to high primary productivity in AB, and that alternative mechanisms that stimulate productivity must be considered.

Spatial patterns further reinforce our hypothesis regarding the role of submesoscale eddies: areas of simulated GMW and BW accumulation correspond to regions of increased observed productivity. The highest mean *Chl-a* values were measured near the mouth of EI, with slightly lower values observed in central AB (Fig. 5e). A significant proportion (82%) of these measurements were preceded by westerly or northwesterly winds, indicating that these locations are particularly conducive to phytoplankton growth under typical westerly wind conditions. The area of increased *Chl-a* in central AB aligns with an enhanced surface anticyclonic circulation cell predicted by the model (Fig. 4g and h). The EI mouth is of particular interest due to its consistently high *Chl-a* values and frequent observations of feeding whales and penguins. This supports our modeling-based conclusions on the importance of areas at the mouths of inner inlets, where wind forcing influences a large portion of the water column and promotes the formation of eddies.

Unfortunately, among the four instances of *in situ* observations conducted on days after periods of easterly or southeasterly winds (Fig. 5a), only one had a sufficiently brief interval from a consecutive measurement day to allow a direct estimate of the impact of a wind direction shift from easterly to westerly on *Chl-a* levels. Specifically, on March 27, 2021 (Fig. 5b), after a period of easterly winds averaging 6.5 m/s, *Chl-a* concentrations were markedly low, with a mean value of 0.1 $\upmu$g/L. During the subsequent five days, the average westerly wind magnitude reached 9.85 m/s. During this period the mean *Chl-a* value increased to 0.4 $\upmu$g/L by April 2 (Fig. 5c), with particularly elevated levels observed in the central region of AB and at the mouth of EI. Although this observation fits our hypothesis, it is based on a single case and thus, obviously, has no statistical significance. However, the satellite data provide a further support for the proposed *Chl-a*–wind relationshp. A Mann–Whitney U-test reveals that GlobColour *Chl-a* anomalies in AB are significantly higher after periods of westerly winds compared to easterly winds (average p = 0.046 for grid points in AB; see “Methods”). This effect appears to be specific to AB, with significant differences observed at 7 of 9 points within the bay (red areas in Fig. 5g). In the broader KGI region, no significant differences were found (p = 0.459 across the area mapped in Fig. 5g), indicating that bay-scale dynamics, rather than larger-scale regional processes, drive the *Chl-a*–wind relationship.

In conclusion, the available observational data, while limited, support our model-derived conjecture regarding the role of cross-bay winds in modulating productivity within Antarctic glacial bays. Our findings suggest that water column stratification alone does not account for the variability in *Chl-a* levels in AB. Regions prone to eddy formation under typical wind conditions exhibit increased productivity, and *Chl-a* values are statistically higher after periods of westerly winds compared to easterly winds. Modeling results show how these disparities can be convincingly explained by changes in AB flow patterns caused by different directions of cross-bay winds. Therefore, the predicted strengthening of westerly winds is expected to enhance AB productivity, while easterly wind events are expected to limit it. We anticipate similar effects in other glacial bays of the WAP, with phytoplankton growth influenced positively or negatively depending on the bay’s orientation relative to the cross-bay wind direction.

## Conclusions

Our research highlights the crucial role of cross-bay winds in shaping circulation patterns within Antarctic glacial bays. In AB westerly winds impede water exchange between the bay and the open ocean and foster formation of submesoscale eddies. The location of these eddies is linked to local geomorphology and is most common near the mouths of the inner inlets, where the Ekman depth $D_E$ approaches the water depth. These eddies can serve as accumulation zones for water masses, including GMW and BW, which are presumed to be key sources of iron in AB. This accumulation is expected to positively influence primary production, potentially leading to the creation of localized biological blooms. In contrast, easterly winds enhance the flushing of AB waters by strengthening the circulation cell in the main body of the bay and significantly increasing the strength of the outflow current. Consequently, easterly winds are presumed to reduce primary production by advecting nutrients and phytoplankton out of the bay.

Using hydrodynamical modeling as its primary tool, this study investigates the significance of a single, previously understudied factor shaping glacial bay circulation: cross-bay winds. It provides critical insights into the potential impacts of strong wind forcing, which would be unattainable through observational methods alone. This is particularly valuable, as wind intensification is projected to increase in the WAP region. The results suggest that wind direction, rather than just wind magnitude, can have significant and far-reaching effects. This study also proposes a possible explanation for how primary production can be maintained in the high-energy coastal waters of the WAP. Future research, integrating coupled hydrodynamical, iron, and biological modeling, could build on this foundation to provide a more comprehensive understanding of glacial bay bloom dynamics and a quantitative assessment of the significance of this mechanism.

The glacial bays of Antarctica are undergoing significant transformation due to climate change. The circumpolar westerly winds are intensifying, the influx of glacial water is increasing, and the extent of sea ice is decreasing. All of these factors influence glacial bay hydrodynamics and primary production. This study represents the first investigation of the direct effects of common cross-bay winds on the hydrodynamics of an Antarctic glacial bay and their potential implications for local primary productivity. We suggest that analogous mechanisms may operate in other similarly shaped bays of WAP where cross-bay winds frequently occur. In a broader context, this study contributes to understanding the impact of ongoing regional transformations on primary production in the coastal regions of the WAP, which is crucial for the food chain and carbon cycle of the entire SO.

## Methods

Wind data were obtained from the Antarctic Mesoscale Prediction Model^20^ (AMPS). The hourly wind direction and magnitude values from seventeen AMPS model grid points from the period December 15, 2018, to March 1, 2023, were collected and spatially averaged. This time range corresponds to the period of the *in situ* measurement campaign conducted in AB, as detailed by Osińska et al.^29^. The *Chl-a* measurements conducted during this campaign used an optical sensor calibrated with deionized water, without laboratory validation of its absolute values. Consequently, all documented *Chl-a* levels are biased by the same value. Both the mean and median *Chl-a* value recorded during winter (from June, July, and August), a season known to have the lowest phytoplankton presence, is −0.44 $\upmu$g/L with a standard deviation of 0.07 $\upmu$g/L. Consequently, −0.44 $\upmu$g/L was taken as an accurate representation of 0 $\upmu$g/L, and all measured values were adjusted accordingly. Days during the winter months (June, July, and August) and days with measurements taken from fewer than 10 sites were excluded from the analyzed dataset (Fig. 5). In the analysis of the relationship between wind forcing and *Chl-a* levels (Fig. 5), AMPS wind data averaged over five days prior to each *Chl-a* measurement day was used. The five-day period was chosen because modeling has shown it to be the maximum time required for the AB circulation pattern to adjust and reach a new equilibrium after a change in wind conditions.

Due to limited availability of high-resolution satellite color imagery for the AB region resulting from persistent cloud cover (for instance, during the over four-year-long sampling campaign, only 16 Sentinel-2 L2A images with less than 15% cloud cover were available from AB) an alternative approach was necessary to assess daily variability in *Chl-a* concentrations. Therefore, daily *Chl-a* estimates from the GlobColour^35^ product were utilized. Data were extracted from a region surrounding KGI (within the spatial limits 63.10–61.73°S, 59.34–57.48°W, *n* = 1466) for the period December 15, 2018, to March 1, 2023. To investigate the potential influence of wind forcing on *Chl-a* levels, a comparative analysis was conducted between days following westerly and easterly wind events (defined as the predominant wind direction during the five days prior to each observation, consistent with the approach used in the measured *Chl-a* data analysis). Initially, *Chl-a* anomalies were calculated to account for seasonal variability. This involved generating a 30-day moving average of *Chl-a* concentrations for each grid point. Subsequently, daily *Chl-a* anomalies were derived by subtracting the smoothed 30-day average from the corresponding daily *Chl-a* value. The *Chl-a* anomaly data were then categorized into three groups: (1) anomalies following easterly wind forcing (predominant wind direction between $45^\circ$ and $135^\circ$), (2) anomalies following westerly wind forcing (predominant wind direction between $225^\circ$ and $315^\circ$), and (3) all remaining data. This classification resulted in 845 *Chl-a* daily values associated with westerly wind events and 167 daily values associated with easterly wind events. A two-sample Mann-Whitney U test was then performed to test the null hypothesis of no significant difference between the *Chl-a* anomaly distributions for the westerly and easterly wind categories. The U-test was chosen since the Shapiro-Wilk test indicated a statistically significant departure from normality for both the easterly and westerly wind condition datasets. This test was applied to each grid point within the GlobColour dataset.

Hydrodynamical modelling was performed using the Delft3D Flow model^38^, following the detailed setup outlined by Osińska et al.^7,38^. Two modifications were made: the resolution of the open boundary condition was improved from three to five data points, and a uniform 3D Chézy bottom roughness coefficient of 50 m^1/2^/s was applied. The analysis of results in this paper is limited to the region outlined by the pink dashed line in Fig. 1b, approximately 5 km from the model’s open boundary. Glacial water influx was introduced uniformly across all glacial fronts, with a volume of 0.64 m$^3$/s per approximately 1 km of glacial front. This corresponds to a total of 19 m$^3$/s, representing the average value of glacial influx to AB during the months of December and January, as estimated by Osińska et al.^7^.

The D-WAQ Part particle tracking model^39^ has been coupled with the hydrodynamical model. The Delft3D Flow model ran with a timestep of 0.06 minutes, and results were recorded every 12 minutes. Based on its results, the particle tracking model was calculated with a 3 minute timestep. The vertical resolution of the D-WAQ Part model was established at 25 layers within the $\sigma$-coordinate system.

The vertical dispersion values fed to D-WAQ Part were calculated by the Delft3D Flow using a $k-\varepsilon$ turbulence model. This approach involves solving transport equations for both turbulent kinetic energy ($k$) and its dissipation rate ($\varepsilon$), from which the mixing length and the vertical eddy viscosity ($A_{V}$) are derived^38^. Despite its widespread use, the $k-\varepsilon$ closure model has several well-known limitations, particularly in small-scale or confined domains in which complex flow patterns are present. The $\varepsilon$-equation assumes locally isotropic small-scale turbulence and is tied to the eddy-viscosity hypothesis, which cannot represent anisotropy, curvature effects, or secondary flows. As a result, horizontal shear, side-wall effects, and small recirculation cells are only indirectly captured^40^. Therefore, in locally constrained parts of AB, such as narrow passages, inner embayments, or regions with sharp bathymetric gradients, the model may produce overly smoothed turbulence fields and not fully resolve finer 3D structures.

Horizontal dispersion values were also calculated using Delft3D Flow; however, in the Delft3D framework, additional horizontal dispersion may be added into Lagrangian model calculations. The additional horizontal dispersion is expressed as $D=at^b$, where *t* denotes particle age. The coefficient *a* represents a constant addition of dispersion, while *b* is the dispersion growth factor. As the goal of this study is to monitor individual tracers rather than collective clouds of particles, we set $b=0$, thus making *a* a total added horizontal dispersion coefficient. Given that horizontal dispersion values are recorded in the results of dynamic turbulence models and utilized in pathway tracking calculations, it is recommended that the supplementary dispersion values remain low, approximately 1 m$^2$/s^39^. There are no direct measurements of dispersion in this area; therefore, we tested three scenarios of additional horizontal dispersion: 0.03 m$^2$/s (estimation based on^41^), 0.07 m$^2$/s (suggested value by the model developer in the example case^39^), and 1 m$^2$/s (the default $a$ value). The testing indicated that, statistically, this value did not significantly influence the results, demonstrating that the hydrodynamical turbulence values and model resolution are sufficient and that this additional coefficient is of lesser importance. The middle value of 0.07 m$^2$/s was used in all subsequent calculations.

Three water masses were monitored in the D-WAQ Part model: GMW, BW, and OB. Tracked particles were released in four groups at distinct moments of the tidal cycle: at maximum high water, during the middle of the ebb tide, at minimum low water, and in the middle of the flood tide. The presented results are averaged across these four groups, thereby reducing the potential impact of the tidal cycle on the model results. For each release point and moment, 150 particles were tracked. GMW water particles were observed from 52 points along the ocean/glacier interface (Fig. 1b), at the bottom, middle, and top of the water column, resulting in a total of 86,400 GMW particles being tracked. BW tracking started from 20 evenly distributed points in the innermost section of the deep trench located in the center of AB, as indicated in Fig. 1b by a purple circle, totaling 12,000 BW water tracers. OB particles were released from the center of the robust inflowing western current in the western section of the open boundary (green dot in Fig. 1b), from 25 depths, contributing to a total of 15,000 tracked OB water pathways. Modeling continued for a duration of 39 days (Dec 1st, 2021–Jan 9th, 2022), which included a 5-day spin-up period. Particle tracking and wind forcing began during day 6.

Calculations of buoyancy frequency *N* were performed using the Gibbs Oceanographic Toolbox function gsw_N$^{\mathtt {\wedge }}$2, divided by $2\pi$ and square-rooted^42^.

The first baroclinic Rossby radius of deformation (*A*) was calculated using the formula^43^:

1 $$\begin{aligned} A=\frac{c}{|f|}, \end{aligned}$$

where *f* is the Coriolis parameter and *c* is the internal wave speed:

2 $$\begin{aligned} c=\frac{1}{\pi }\int _{-H}^{0} N(z)dz. \end{aligned}$$

For each grid point *i* within the inner domain (outlined by the pink dashed line in Fig. 1b), the maximum value of $A_{V}$ was extracted from its vertical profile at every timestep. The $A_{Vi}$ was then obtained as the time-average of these maximum values over the 33-day simulation. Subsequently Ekman layer depth ($D_E$) was calculated using a simple formula^44^:

3 $$\begin{aligned} D_E=\frac{1}{n}\sum _{i=1}^n{\pi \sqrt{\frac{2A_{Vi}}{|f|}}}. \end{aligned}$$

The vorticity was calculated as the curl of the horizontal velocity field, averaged through 33 days of modeling.

## Supplementary Information

Supplementary Information 1.

Supplementary Information 2.

## Data Availability

The modelling setup files and results are available from the corresponding author on reasonable request. The observational data analyzed during the current study can be found in online repositories: PANGAEA - https://doi.org/10.1594/PANGAEA.947909; Zenodo - https://zenodo.org/records/10277429

## References

[1] Ducklow, H. W. et al. Marine pelagic ecosystems: The West Antarctic Peninsula. *Philos. Trans. R. Soc. B Biol. Sci.* **362**, 67–94. 10.1098/rstb.2006.1955 (2007). 10.1098/rstb.2006.1955 PMC1764834 17405208

[2] de Baar, H. J., Bathmannt, U., Smetacek, V., Löscher, B. M. & Veth, C. Importance of iron for plankton blooms and carbon dioxide drawdown in the Southern Ocean. *Nature*10.1038/373412a0 (1995).

[3] Boyd, P. W., Arrigo, K. R., Strzepek, R. & van Dijken, G. L. Mapping phytoplankton iron utilization: Insights into Southern Ocean supply mechanisms. *J. Geophys. Res. Oceans*10.1029/2011JC007726 (2012).

[4] Annett, A. L. et al. Comparative roles of upwelling and glacial iron sources in Ryder Bay, coastal western Antarctic Peninsula. *Mar. Chem.*10.1016/j.marchem.2015.06.017 (2015).

[5] De Jong, J. et al. Natural iron fertilization of the Atlantic sector of the Southern Ocean by continental shelf sources of the Antarctic Peninsula. *J. Geophys. Res. Biogeosci.*10.1029/2011JG001679 (2012).

[6] Jones, R. L. et al. Antarctic glaciers export carbon-stabilised iron(II)-rich particles to the surface Southern Ocean. *Nat. Commun.* **16**, 1–10. 10.1038/s41467-025-59981-y (2025). 40447594 10.1038/s41467-025-59981-y PMC12125279

[7] Osińska, M. & Herman, A. Influence of glacial influx on the hydrodynamics of Admiralty Bay, Antarctica - study based on combined hydrographic measurements and numerical modeling. *Front. Mar. Sci.* **11**, 1365157. 10.3389/FMARS.2024.1365157 (2024).

[8] Schloss, I. R. et al. On the phytoplankton bloom in coastal waters of southern King George Island (Antarctica) in January 2010: An exceptional feature?. *Limnol. Oceanogr.* **59**, 195–210. 10.4319/lo.2014.59.1.0195 (2014).

[9] Höfer, J. et al. The role of water column stability and wind mixing in the production/export dynamics of two bays in the Western Antarctic Peninsula. *Progr. Oceanogr..*10.1016/j.pocean.2019.01.005 (2019).

[10] Zhao, K. X., Stewart, A. L., McWilliams, J. C., Fenty, I. G. & Rignot, E. J. Standing eddies in glacial Fjords and their role in Fjord circulation and melt. *J. Phys. Oceanogr.* **53**, 821–840. 10.1175/JPO-D-22-0085.1 (2023).

[11] Neder, C. et al. Modelling suspended particulate matter dynamics at an Antarctic fjord impacted by glacier melt. *J. Mar. Syst.* **231**, 103734. 10.1016/J.JMARSYS.2022.103734 (2022).

[12] Sandulescu, M., López, C., Hernández-García, E. & Feudel, U. Plankton blooms in vortices: The role of biological and hydrodynamic timescales. *Nonlinear Process. Geophys.*10.5194/npg-14-443-2007 (2007).

[13] Cushman-Roisin, B., Asplin, L. & Svendsen, H. Upwelling in broad fjords. *Continental Shelf Res.*10.1016/0278-4343(94)90044-2 (1994).

[14] Lundesgaard, Ø., Powell, B., Merrifield, M., Hahn-Woernle, L. & Winsor, P. Response of an Antarctic Peninsula Fjord to Summer Katabatic Wind Events. *J. Phys. Oceanogr.* **49**, 1485–1502. 10.1175/JPO-D-18-0119.1 (2019).

[15] Davison, B. J., Hogg, A. E., Moffat, C., Meredith, M. P. & Wallis, B. J. Widespread increase in discharge from west Antarctic Peninsula glaciers since 2018. *Cryosphere* **18**, 3237–3251. 10.5194/tc-18-3237-2024 (2024).

[16] Rakusa-Suszczewski, S. The past and present of King George Island (South Shetland Islands, Antarctica). *Polish Polar Res.* **19**, 249–252 (1998).

[17] Wasiłowska, A., Tatur, A. & Rzepecki, M. Massive diatom bloom initiated by high winter sea ice in Admiralty Bay (King George Island, South Shetlands) in relation to nutrient concentrations in the water column during the 2009/2010 summer. *J. Mar. Syst.* **226**, 103667. 10.1016/j.jmarsys.2021.103667 (2022).

[18] Kanamitsu, M. et al. NCEP-DOE AMIP-II reanalysis (R-2). *Bull. Am. Meteorol. Soc.*10.1175/bams-83-11-1631 (2002).

[19] Gerrish, L., Fretwell, P. & Cooper, P. High resolution vector polylines of the Antarctic coastline (7.4) [Data set]. Tech. Rep., UK Polar Data Centre. *Natural Environment Research Council, UK Research & Innovation*10.5285/e46be5bc-ef8e-4fd5-967b-92863fbe2835 (2021).

[20] Powers, J. G. et al. Real-time mesoscale modeling over Antarctica: The Antarctic mesoscale prediction system. *Bull. Am. Meteorol. Soc.*10.1175/BAMS-84-11-1533 (2003).

[21] Wójcik-Długoborska, K. A., Osińska, M. & Bialik, R. J. The impact of glacial suspension color on the relationship between its properties and marine water spectral reflectance. *IEEE J. Sel. Topics Appl. Earth Observ. Remote Sens.*10.1109/JSTARS.2022.3166398 (2022).

[22] Sierpinski, S., Baquer, L. M., Martins, C. C. & Grassi, M. T. Exploratory evaluation of iron and its speciation in surface waters of Admiralty Bay, King George Island, Antarctica. *Anais da Academia Brasileira de Ciencias*10.1590/0001-3765202320211520 (2023). 37585980 10.1590/0001-3765202320211520

[23] Klunder, M. B., Laan, P., Middag, R., Baar, H. J. D. & van Ooijen, J. C. Dissolved iron in the Southern Ocean (Atlantic sector). *Deep Sea Res. Part II* **58**, 2678–2694. 10.1016/J.DSR2.2010.10.042 (2011).

[24] Brandini, F. P. & Rebello, J. Wind field effect on hydrography and chlorophyll dynamics in the coastal pelagial of Admiralty Bay, King George Island, Antarctica. *Antarctic Sci.*10.1017/s0954102094000672 (1994).

[25] Plenzler, J., Budzik, T., Puczko, D. & Bialik, R. J. Climatic conditions at arctowski station (king george island, west antarctica) in 2013–2017 against the background of regional changes. *Polish Polar Res.* **40, 1–27**, 10.24425/PPR.2019.126345 (2019).

[26] National Snow and Ice Data Center & CIRES. Sea Ice Index (2023).

[27] Turner, J. et al. Strong wind events in the Antarctic. *J. Geophys. Res. Atmos.*10.1029/2008JD011642 (2009).

[28] Swart, N. C. & Fyfe, J. C. Observed and simulated changes in the Southern Hemisphere surface westerly wind-stress. *Geophys. Res. Lett.*10.1029/2012GL052810 (2012).

[29] Osińska, M., Wójcik-Długoborska, K. A. & Bialik, R. J. Annual hydrographic variability in Antarctic coastal waters infused with glacial inflow. *Earth Syst. Sci. Data* **15**, 607–616. 10.5194/essd-15-607-2023 (2023).

[30] IOC, SCOR & IAPSO. The international thermodynamic equation of seawater – 2010: Calculation and use of thermodynamic properties. *Intergovernmental Oceanographic Commission, Manuals and Guides No. 56* (2010).

[31] Monsen, N. E., Cloern, J. E., Lucas, L. V. & Monismith, S. G. A comment on the use of flushing time, residence time, and age as transport time scales. *Limnol. Oceanogr.*10.4319/lo.2002.47.5.1545 (2002).

[32] Castelao, R. M., Dinniman, M. S., Amos, C. M., Klinck, J. M. & Medeiros, P. M. Eddy-Driven Transport of Particulate Organic Carbon-Rich Coastal Water Off the West Antarctic Peninsula. *J. Geophys. Res. Oceans.*10.1029/2020JC016791 (2021).

[33] Kahru, M., Mitchell, B. G., Gille, S. T., Hewes, C. D. & Holm-Hansen, O. Eddies enhance biological production in the Weddell-Scotia Confluence of the Southern Ocean. *Geophys. Res. Lett.*10.1029/2007GL030430 (2007).

[34] Huot, Y. et al. Does chlorophyll a provide the best index of phytoplankton biomass for primary productivity studies? *Biogeosciences Discussions* (2007).

[35] (CMEMS), E. C. M. S. I. Global ocean colour (copernicus-globcolour), bio-geo-chemical, l4 (monthly and interpolated) from satellite observations (1997-ongoing). 10.48670/moi-00281.

[36] Lipski, M. Variations of physical conditions, nutrients and chlorophyll a contents in Admiralty Bay (King George Island, South Shetland islands, 1979). *Polish Polar Res.* **8**, 307–332 (1987).

[37] Schloss, I. R. et al. Response of phytoplankton dynamics to 19-year (1991–2009) climate trends in Potter Cove (Antarctica). *J. Mar. Syst.* **92**, 53–66. 10.1016/j.jmarsys.2011.10.006 (2012).

[38] Deltares. Delft3D 3D/2D modelling suite for integral water solutions Hydro-Morphodynamics (2020).

[39] Deltares. D-WAQ PART - User Manual (2024).

[40] Pope, S. B. *Turbulent Flows* (Cambridge University Press, 2000).

[41] Neumann, D., Callies, U. & Matthies, M. Marine litter ensemble transport simulations in the southern North Sea. *Mar. Pollut. Bull.* **86**, 219–228. 10.1016/J.MARPOLBUL.2014.07.016 (2014). 25125287 10.1016/j.marpolbul.2014.07.016

[42] McDougall, P., Trevor J. ; Barker. Getting started with TEOS-10 and the Gibbs Seawater (GSW) Oceanographic Toolbox. *Scor/Iapso Wg127* (2011).

[43] Chelton, D. B. et al. Geographical variability of the first baroclinic Rossby radius of deformation. *J. Phys. Oceanogr.* **28**, 433–460 (1998).

[44] Olbers, D., Willebrand, J. & Eden, C. *Ocean dynamics* Vol. 9783642234507 (Springer, 2012).

[45] Padman, L., Fricker, H. A., Coleman, R., Howard, S. & Erofeeva, L. A new tide model for the Antarctic ice shelves and seas. *Ann. Glaciol.* **34**, 247–254. 10.3189/172756402781817752 (2002).

[46] Dotto, T. S., Mata, M. M., Kerr, R. & Garcia, C. A. A novel hydrographic gridded data set for the northern Antarctic Peninsula. *Earth Syst. Sci. Data* **13**, 671–696. 10.5194/ESSD-13-671-2021 (2021).

