# Supplementary material for: Hydrodynamic response of an Antarctic glacial bay to cross-bay winds and its potential impact on primary production
Source: Sci Rep. 2026 Jan 30;16:4354. doi: 10.1038/s41598-025-34031-1 (PMC12865181; doi:10.1038/s41598-025-34031-1)
Supplement: Supplementary file 1 — Supplementary Information 1. [file 41598_2025_34031_MOESM1_ESM.pdf]

Supplementary Information

Hydrodynamic response to cross-bay winds in an Antarctic glacial bay and its potential impact on primary production

Maria Osińska[1,2],* and Agnieszka Herman[2]

[1]University of Gdańsk, Faculty of Oceanography and Geography, Gdańsk, 80-309, Poland

[2]Institute of Oceanology of Polish Academy of Sciences, Sopot, 81-712, Poland

*corresponding.author: maria.osinska@phdstud.ug.edu.pl

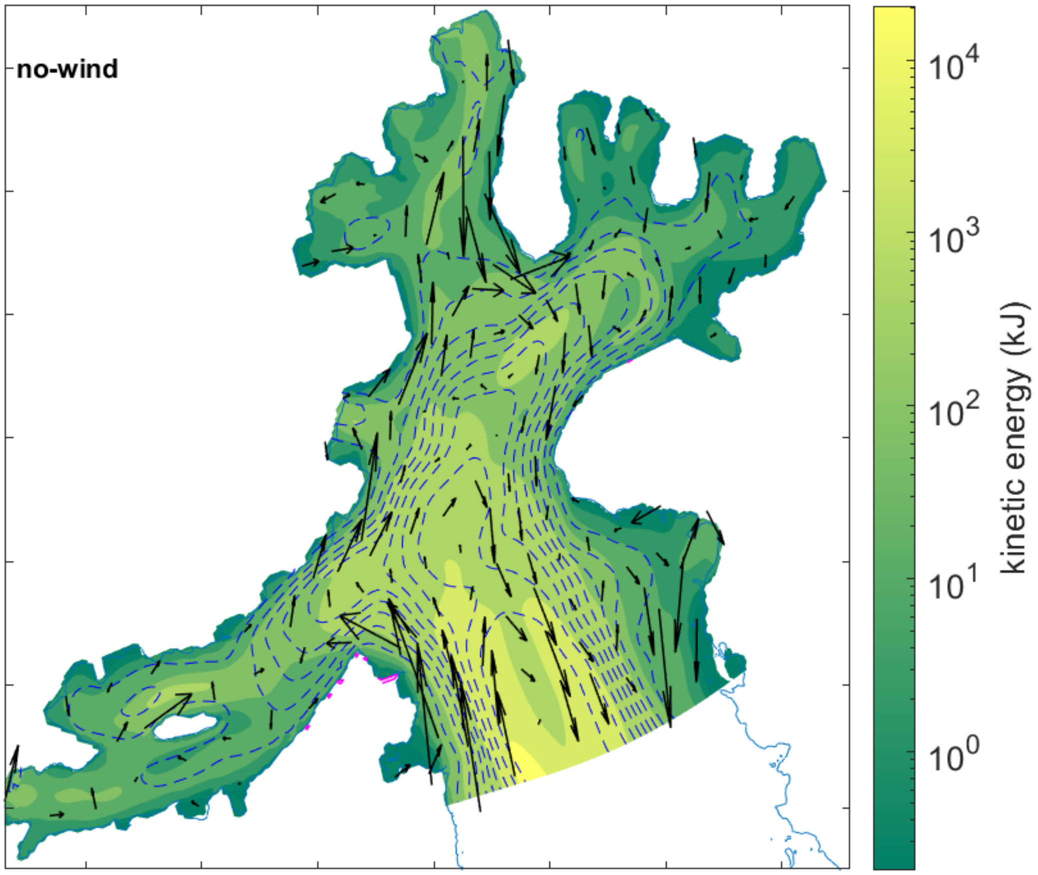

Figure S1. *No-wind scenario kinetic energy and depth averaged velocities; colors represent kinetic energy integrated across the water column; arrows indicate depth-averaged horizontal velocity vectors; blue spaced lines show isobaths, with a magenta line highlighting the isobath = D_E. {Note}: showing mean values from Dec 7, 2021, to Jan 9, 2022 (33 days).*

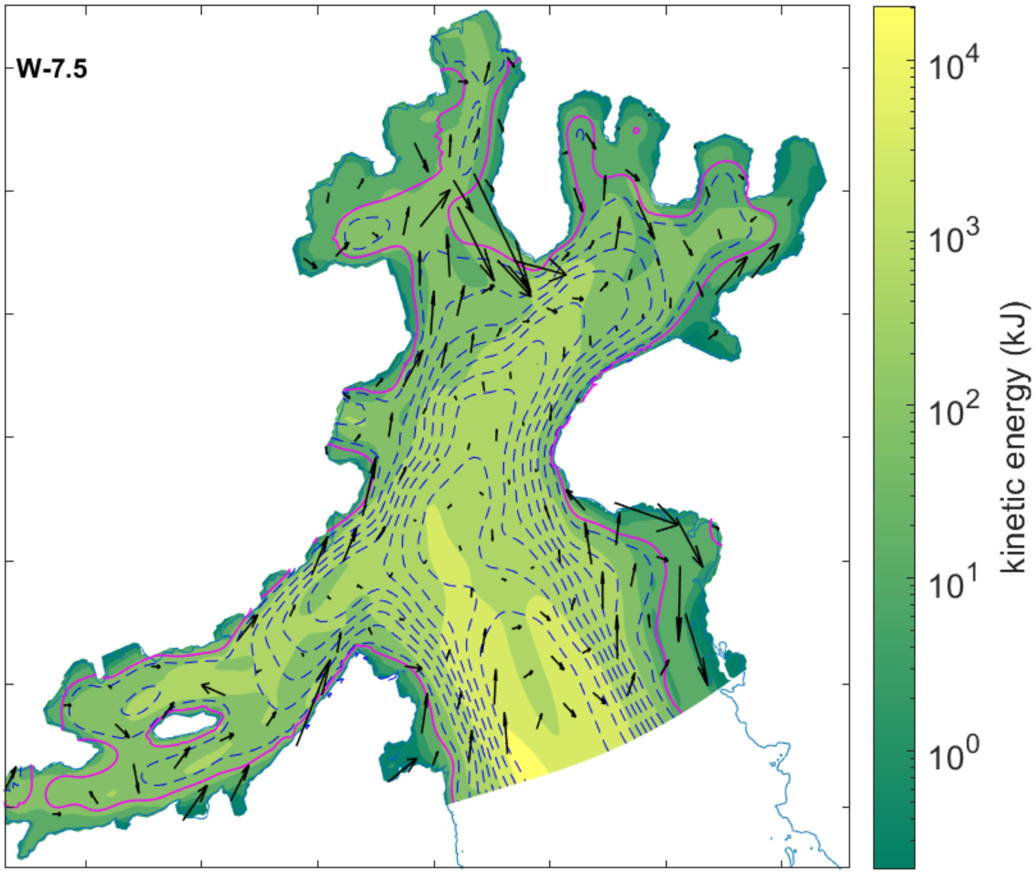

Figure S2. *As in Fig. S1, but for W-7.5 scenario.*

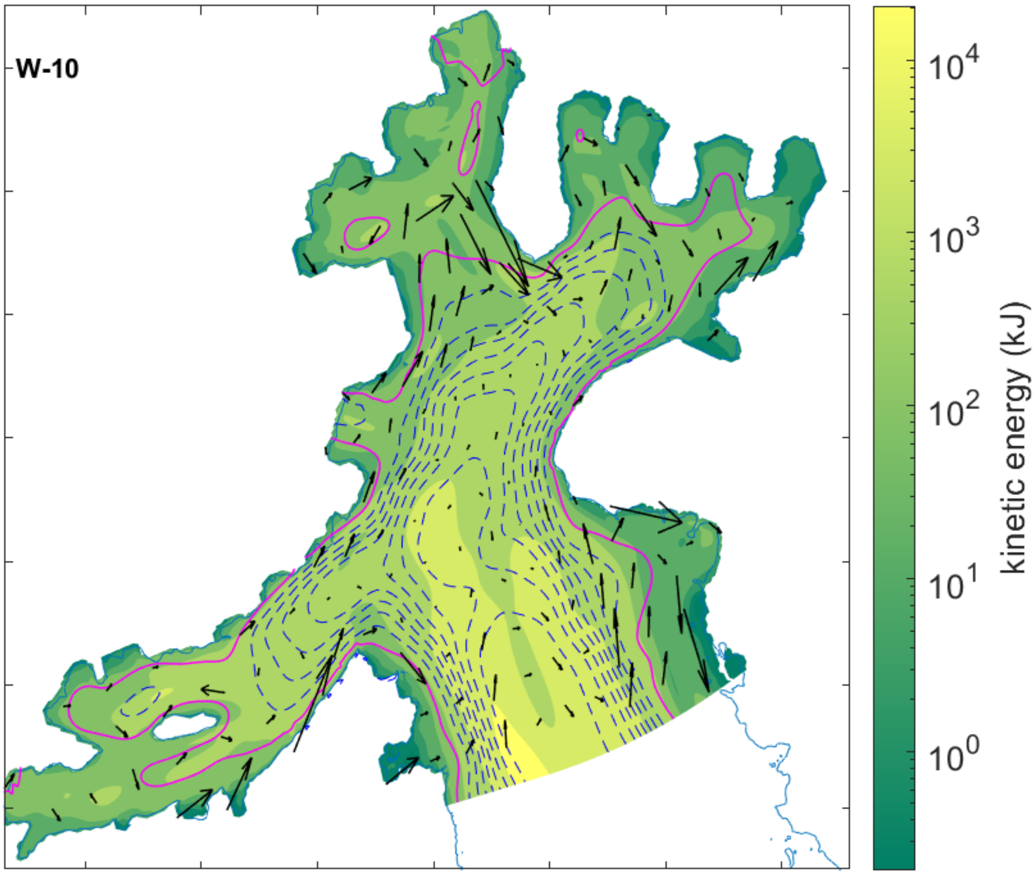

Figure S3. *As in Fig. S1, but for W-10 scenario.*

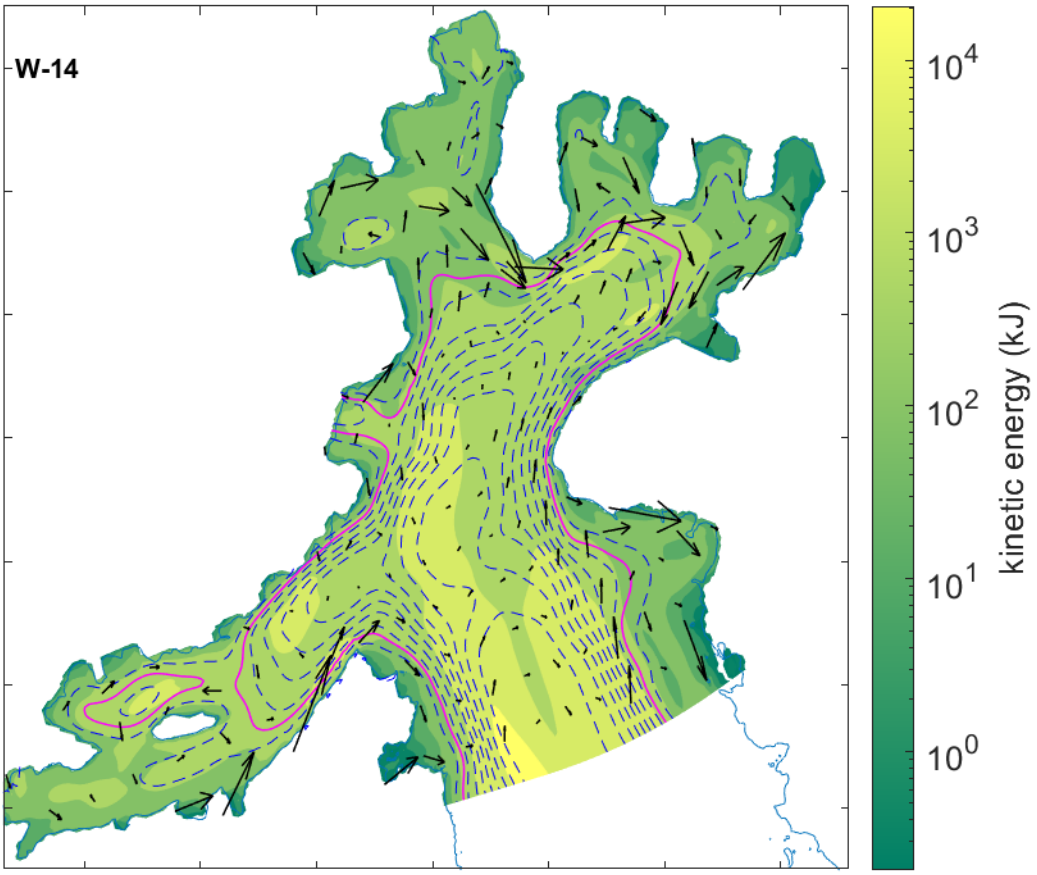

Figure S4. *As in Fig. S1, but for W-14 scenario.*

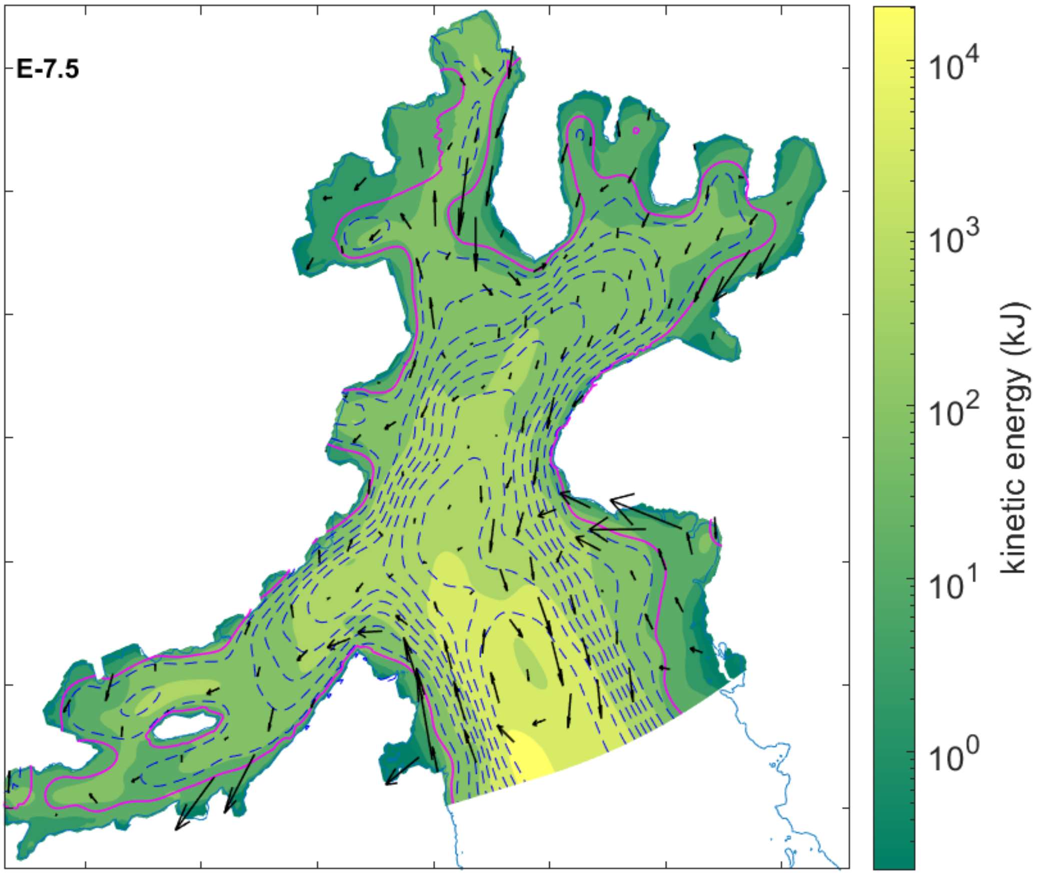

Figure S5. *As in Fig. S1, but for E-7.5 scenario.*

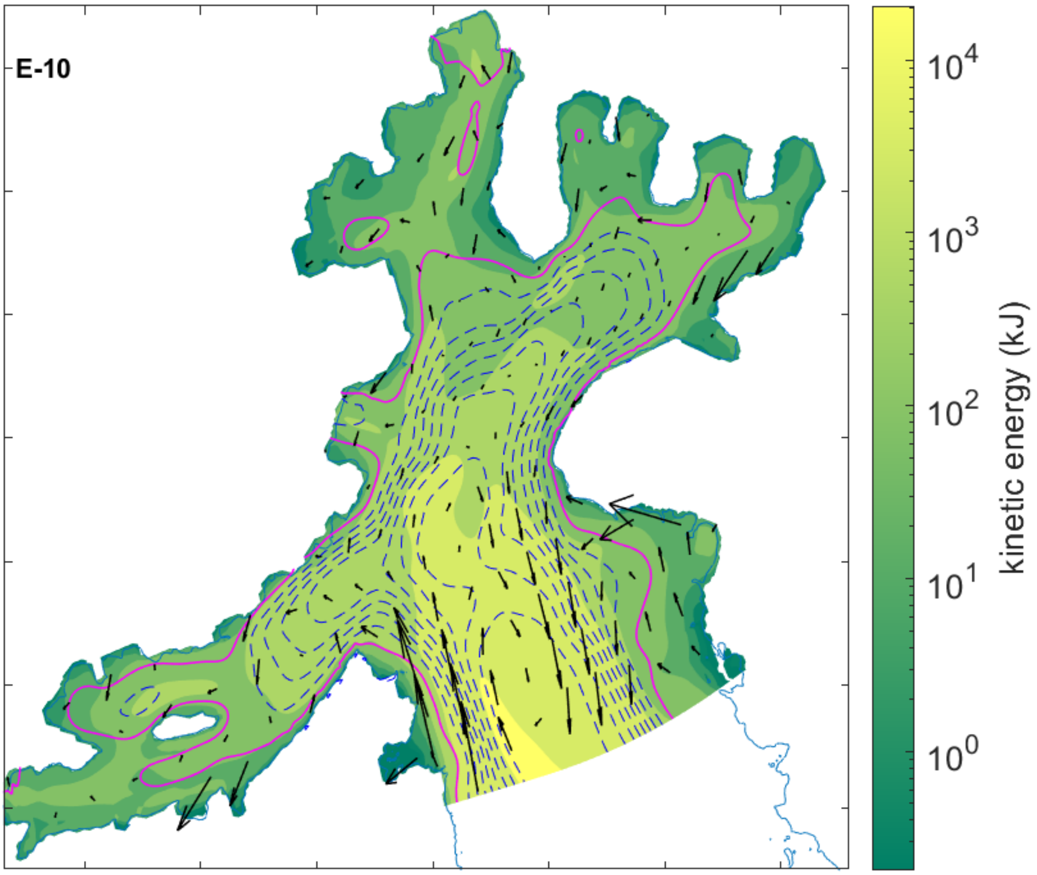

Figure S6. *As in Fig. S1, but for E-10 scenario.*

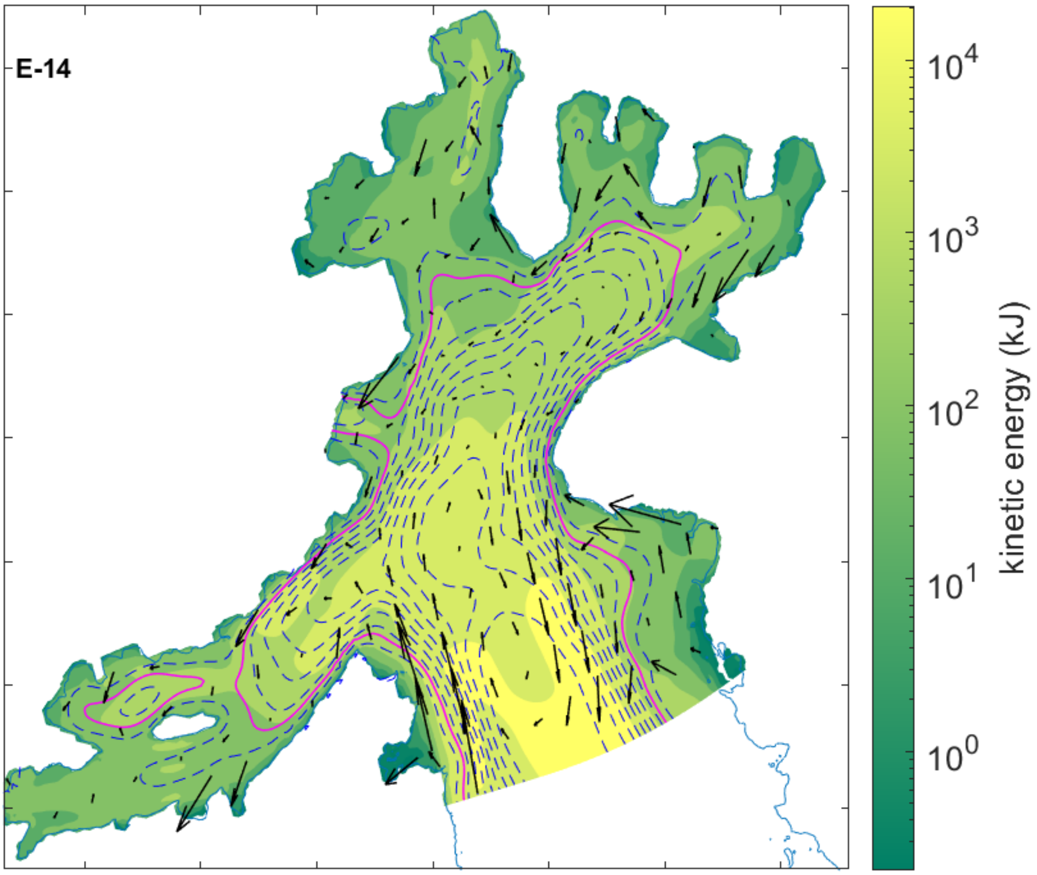

Figure S7. *As in Fig. S1, but for E-14 scenario.*

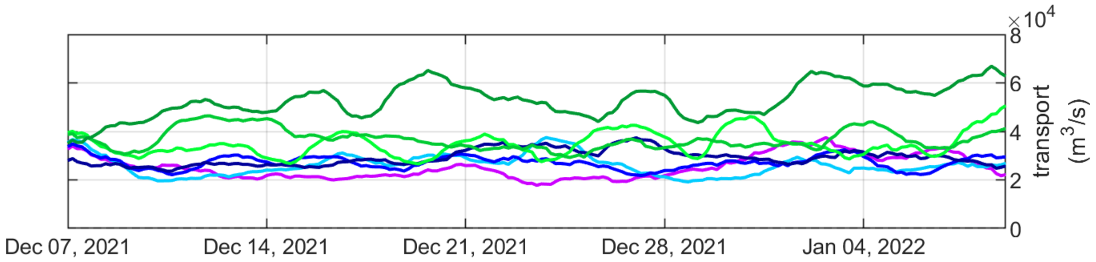

Figure S8*. Volume of transport through the cross-section in the main AB (pink line in Fig. 1 b) in seven model scenarios.*

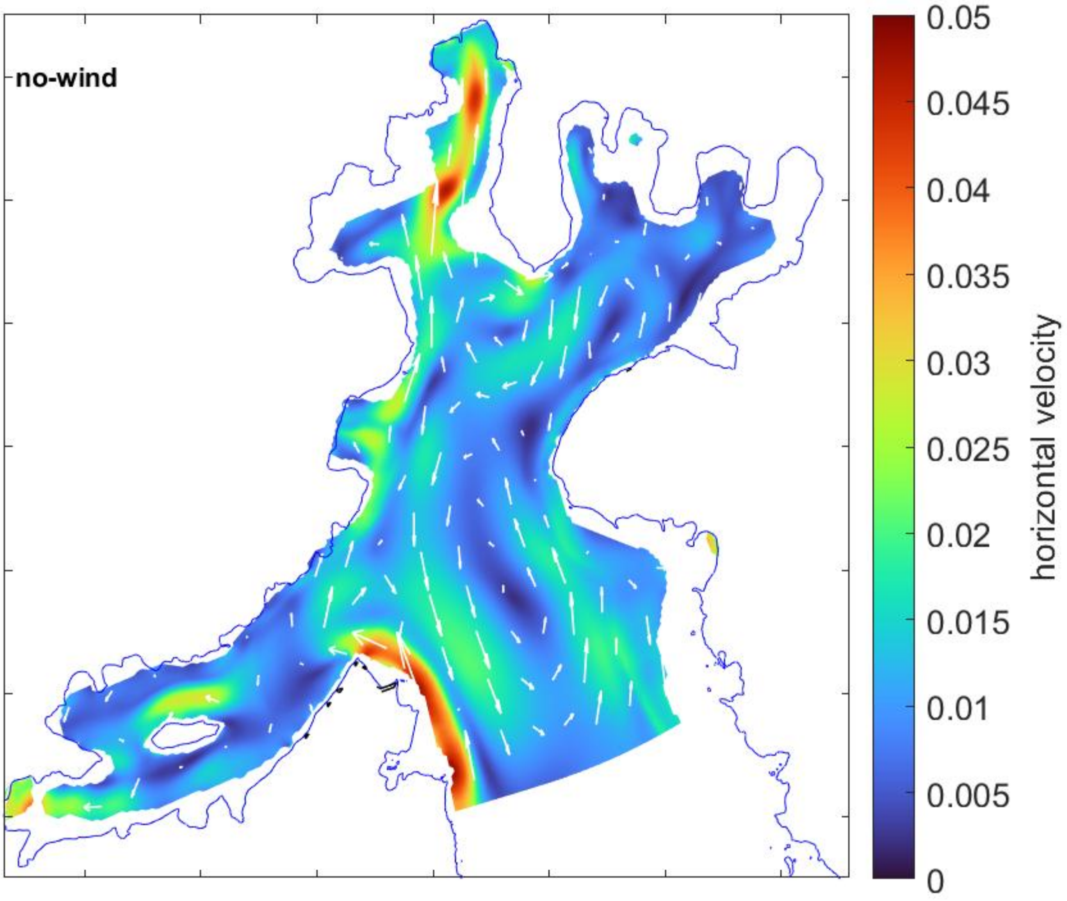

Figure S9. *No-wind scenario horizontal velocities averaged across 10-100 m depth, with a black line highlighting the isobath =* D_E*. {Note}: showing mean values from Dec 7, 2021, to Jan 9, 2022 (33 days).*

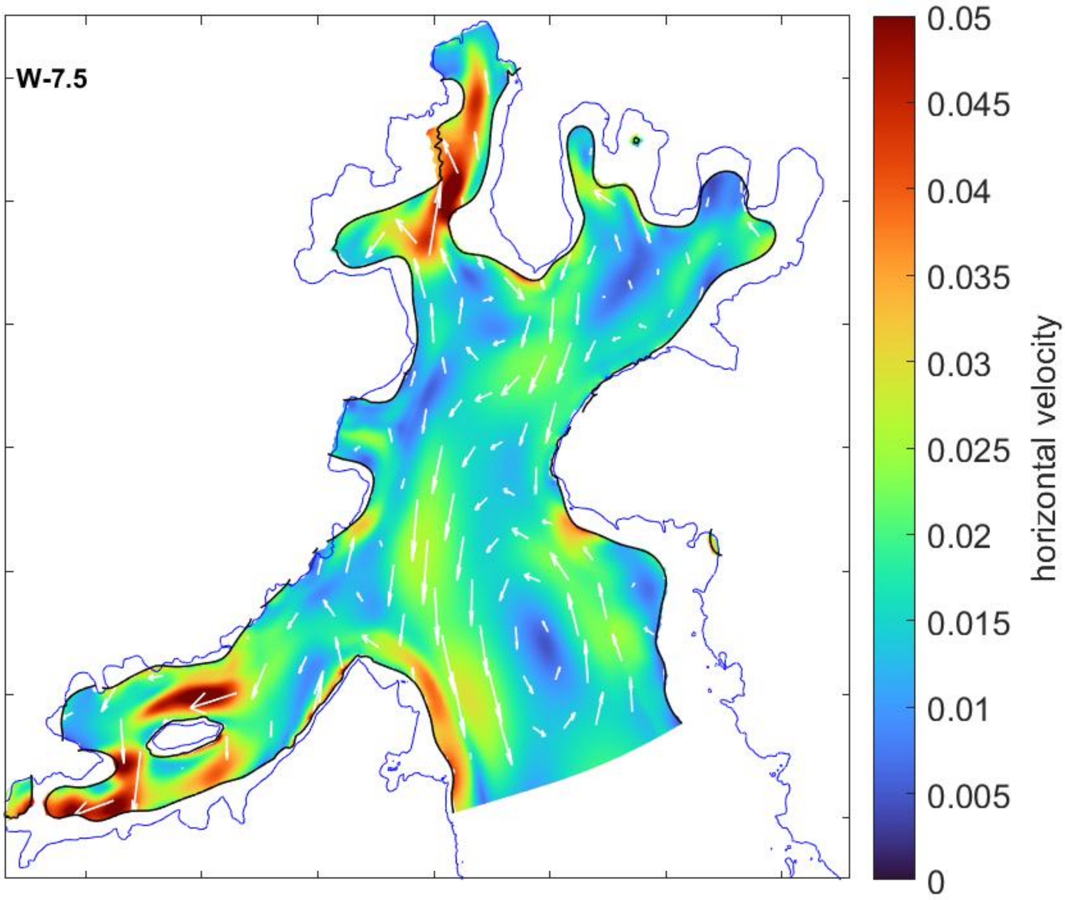

Figure S10. *As in Fig. S9, but for W-7.5 scenario.*

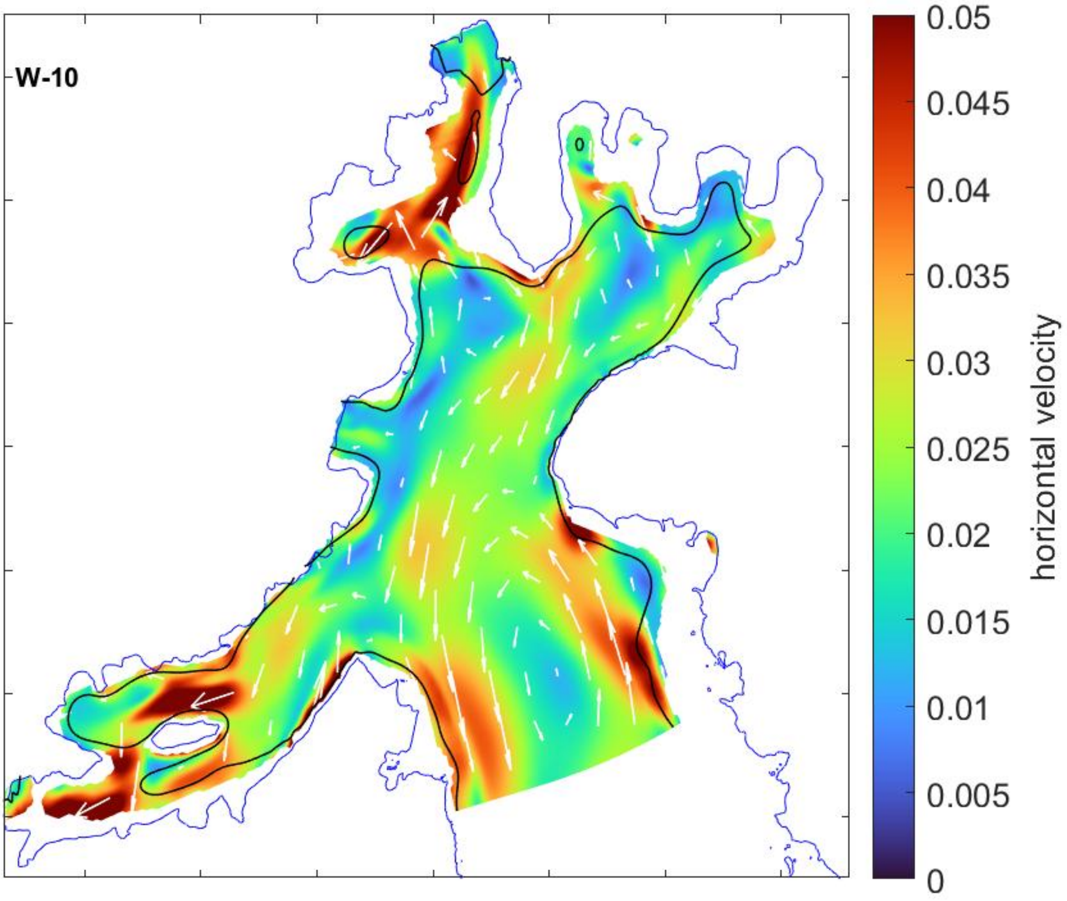

Figure S11. *As in Fig. S9, but for W-10 scenario.*

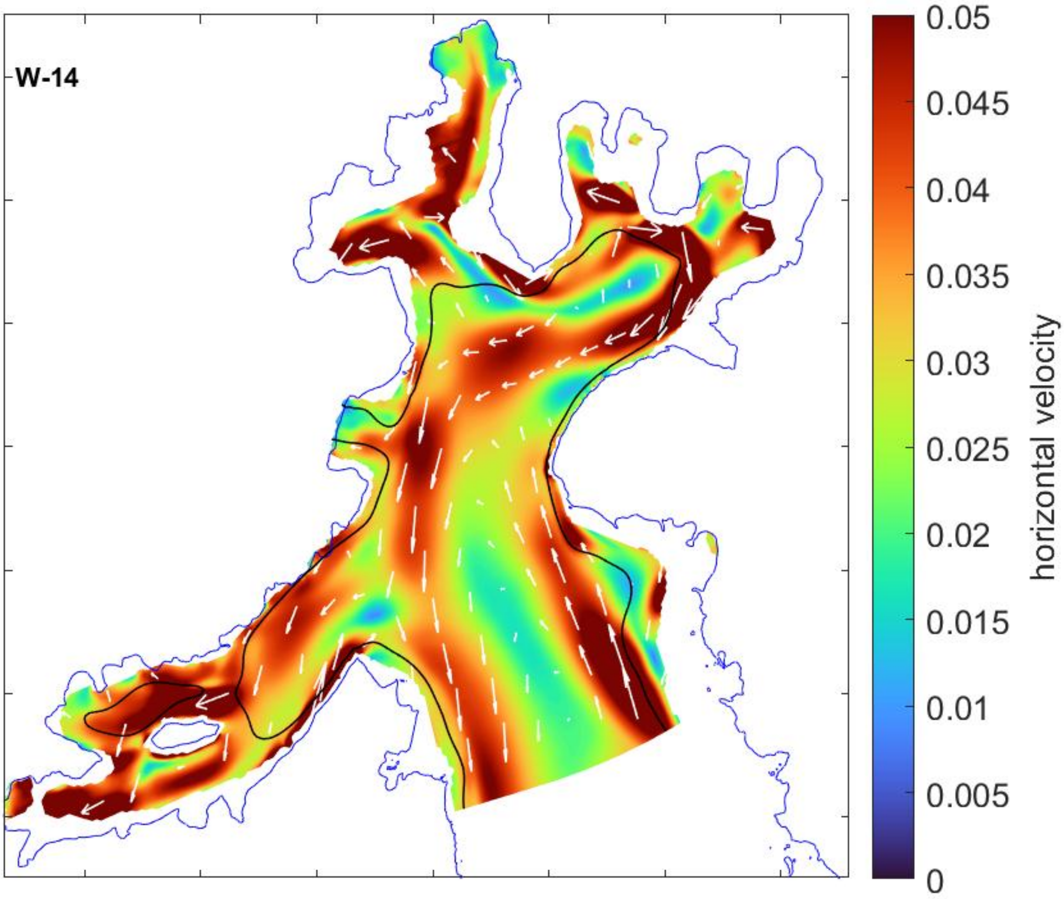

Figure S12. *As in Fig. S9, but for W-14 scenario.*

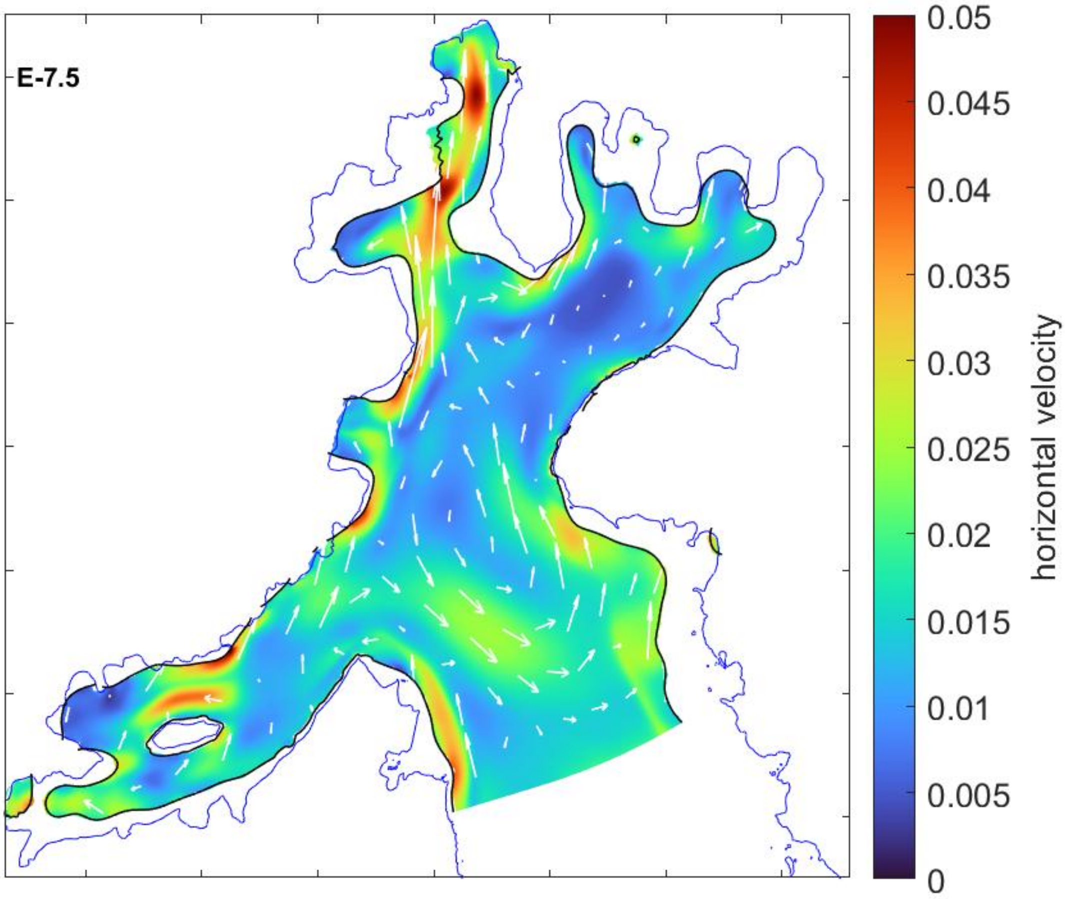

Figure S13. *As in Fig. S9, but for E-7.5 scenario.*

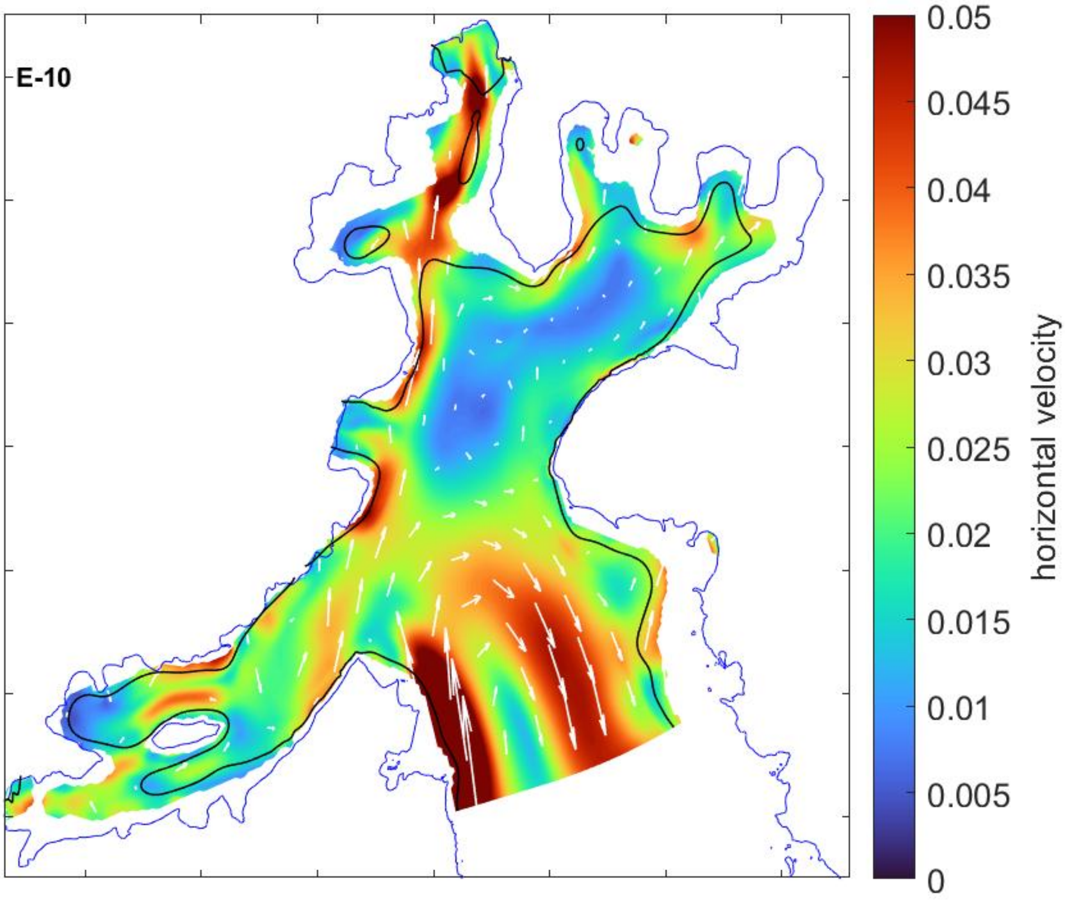

Figure S14. *As in Fig. S9, but for E-10 scenario.*

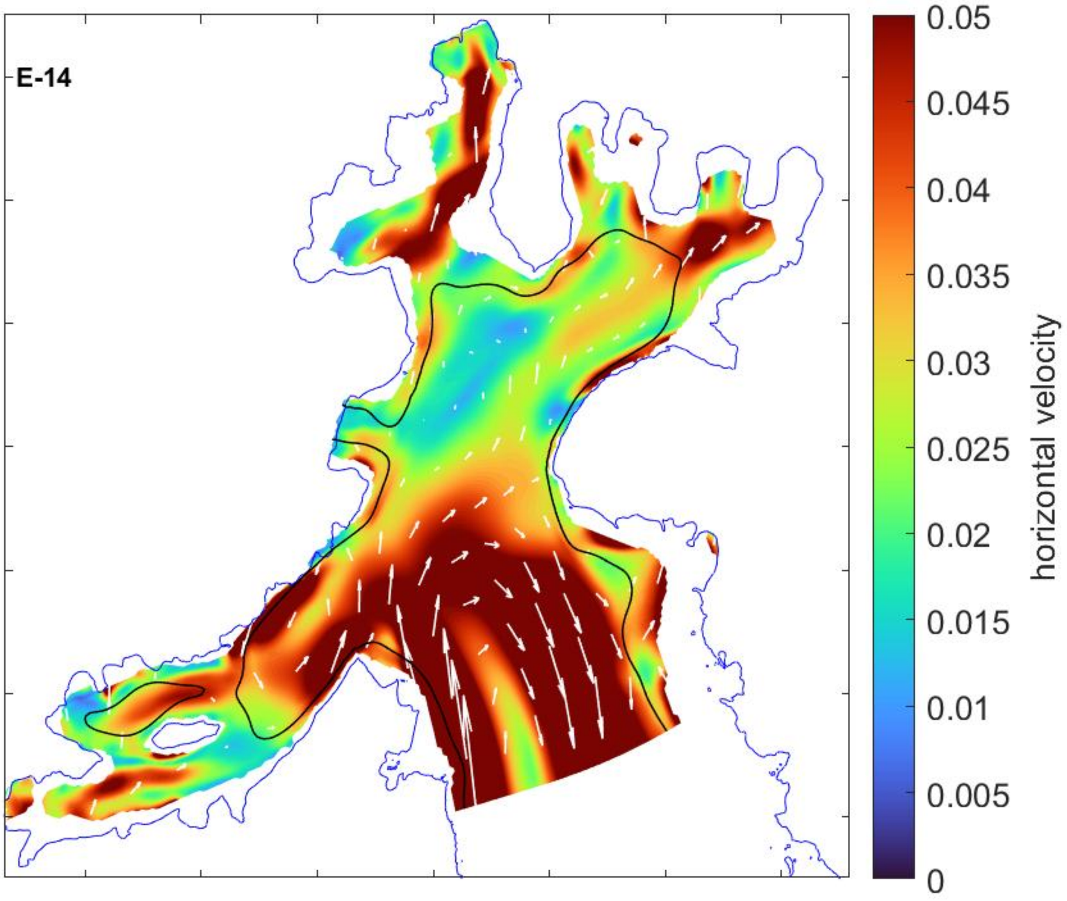

Figure S15. *As in Fig. S9, but for E-14 scenario.*

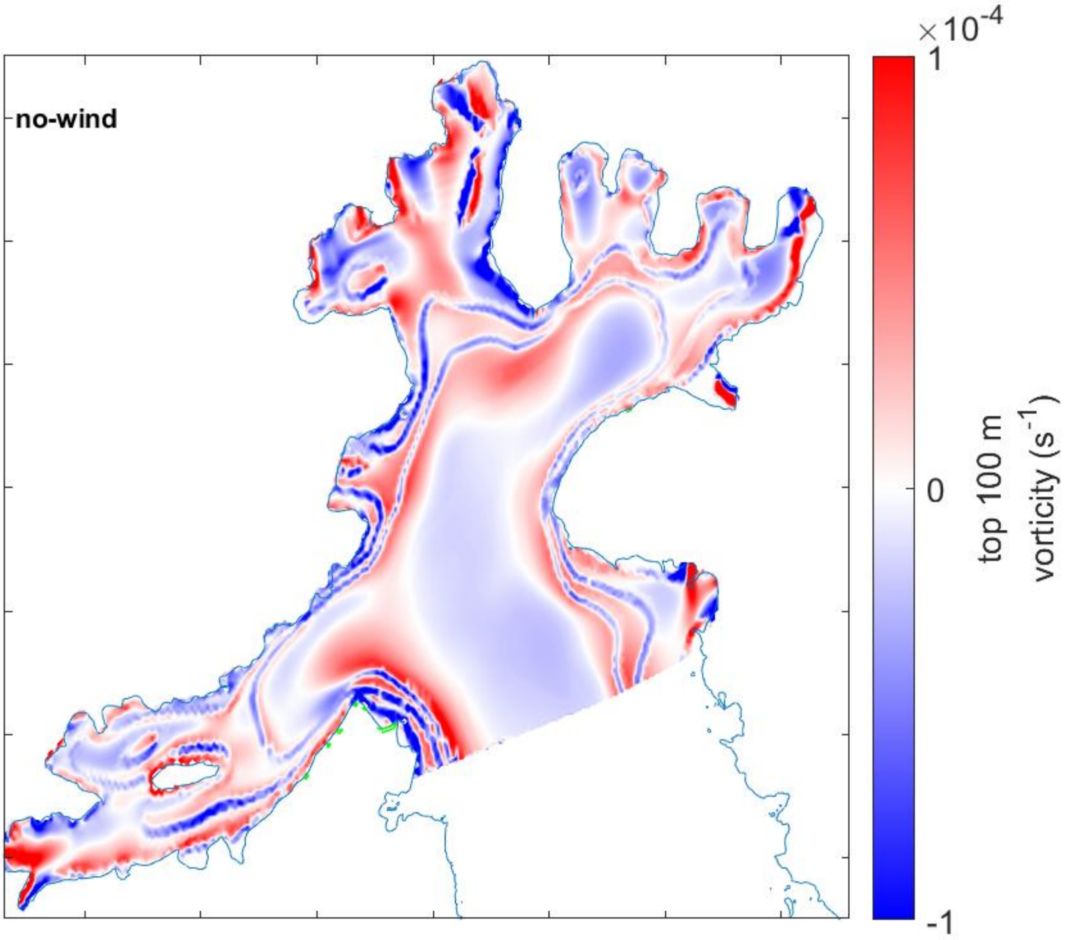

Figure S16. *No-wind vorticity across 10-100 m depth. {Note}: showing mean values from Dec 7, 2021, to Jan 9, 2022 (33 days).*

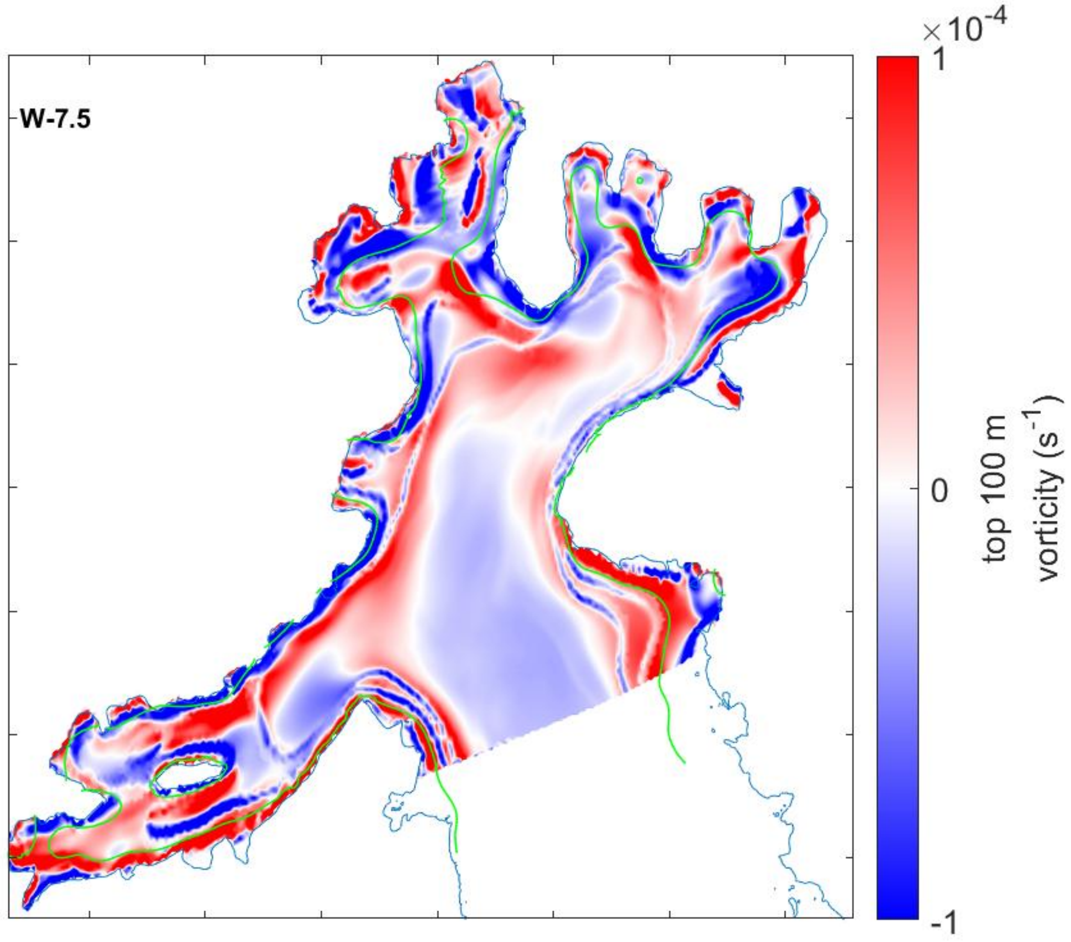

Figure S17. *As in Fig. S16, but for W-7.5 scenario.*

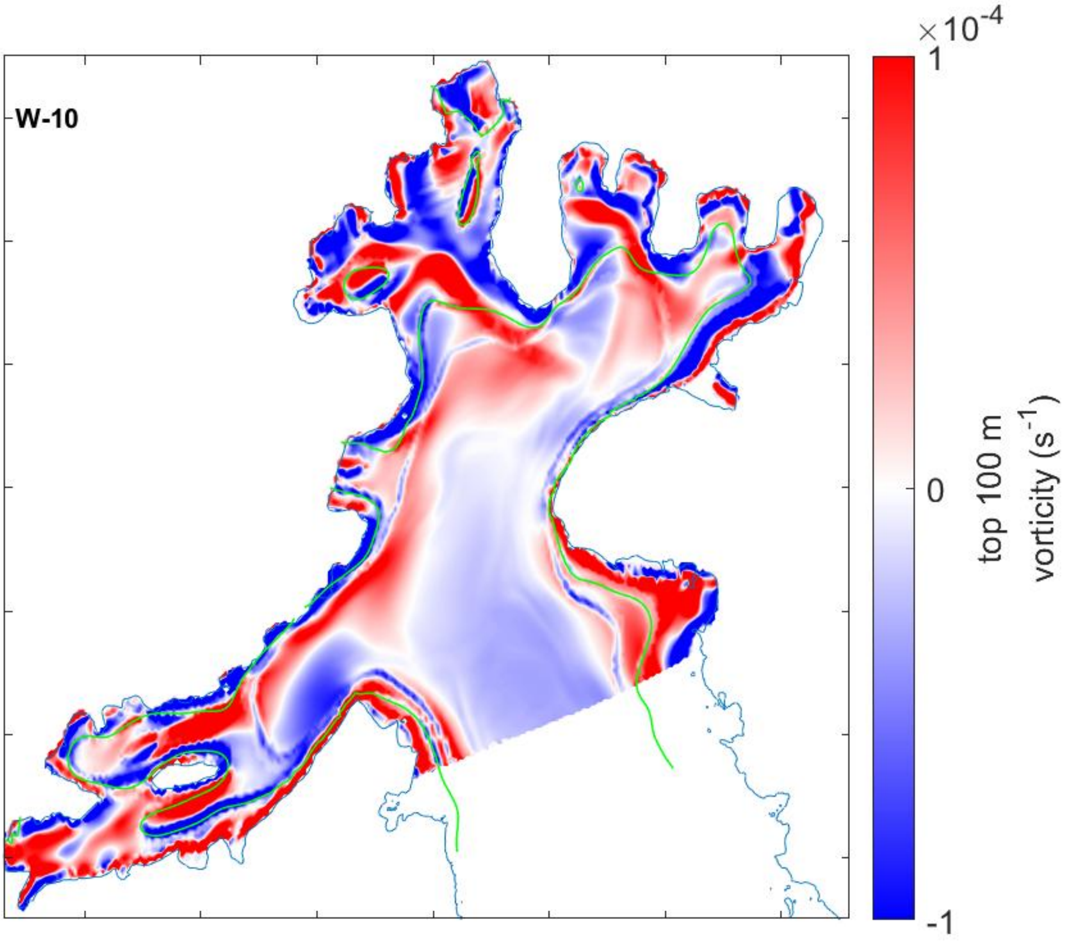

Figure S18. *As in Fig. S16, but for W-10 scenario.*

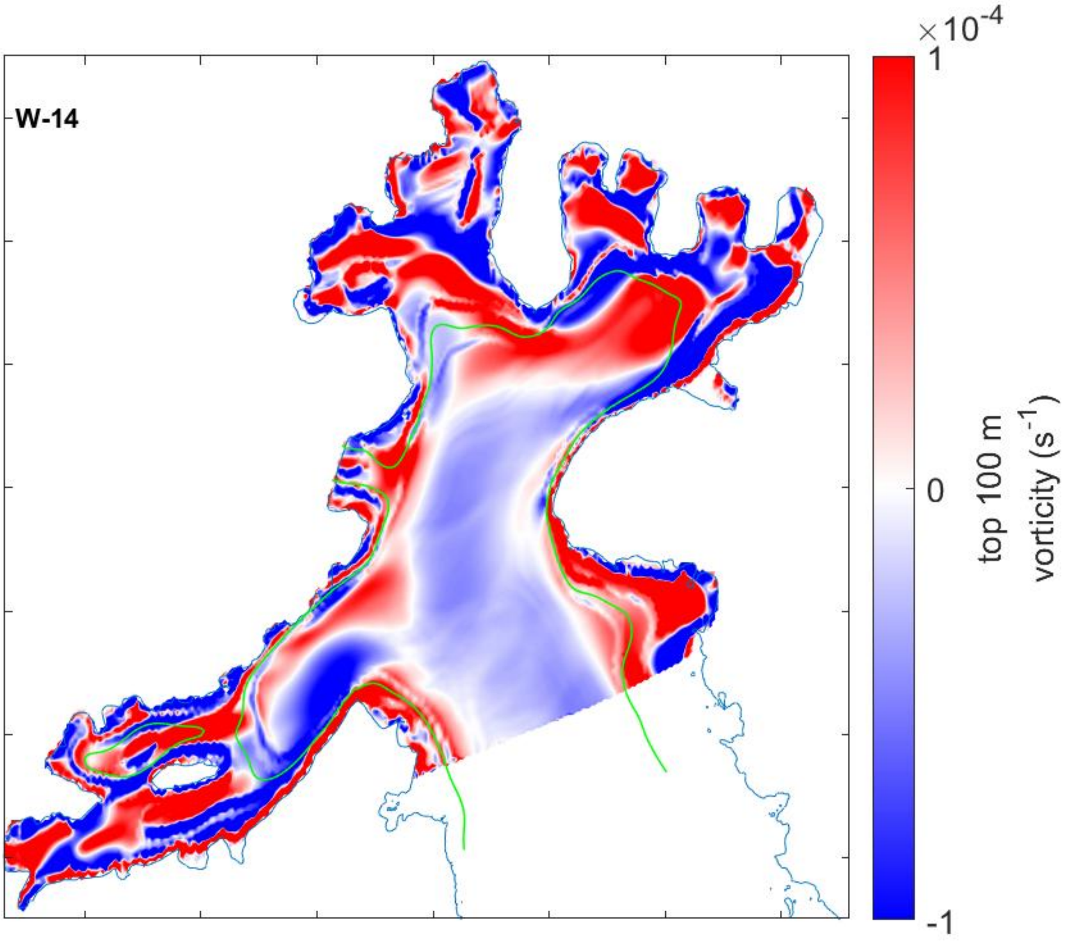

Figure S19. *As in Fig. S16, but for W-14 scenario.*

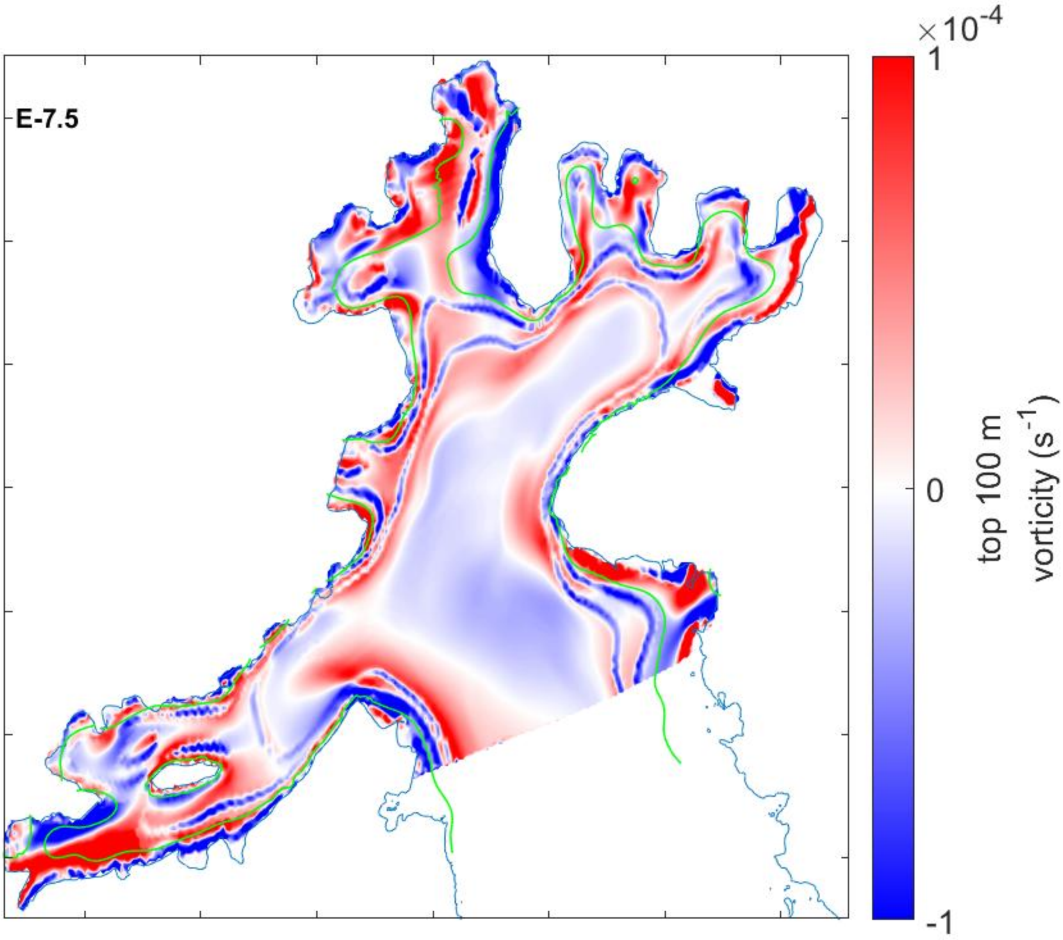

Figure S20. *As in Fig. S16, but for E-7.5 scenario.*

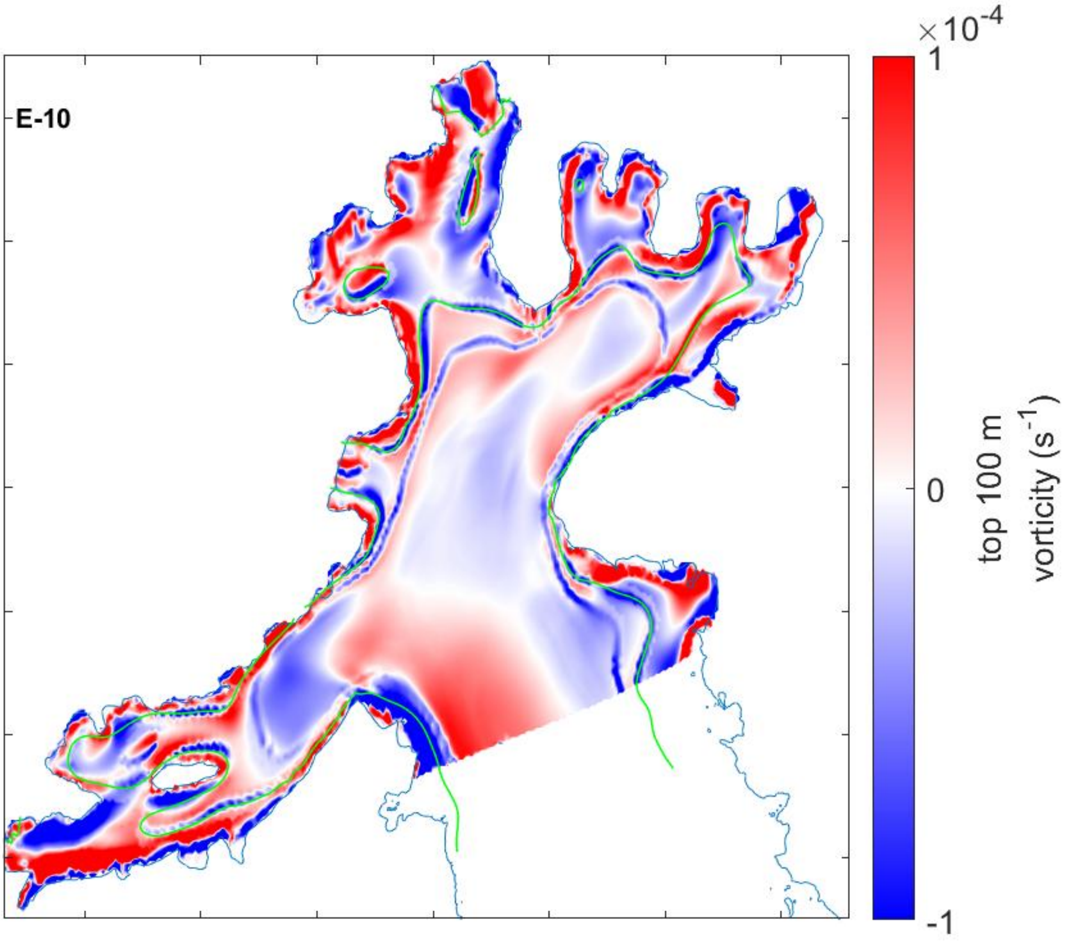

Figure S21. *As in Fig. S16, but for E-10 scenario.*

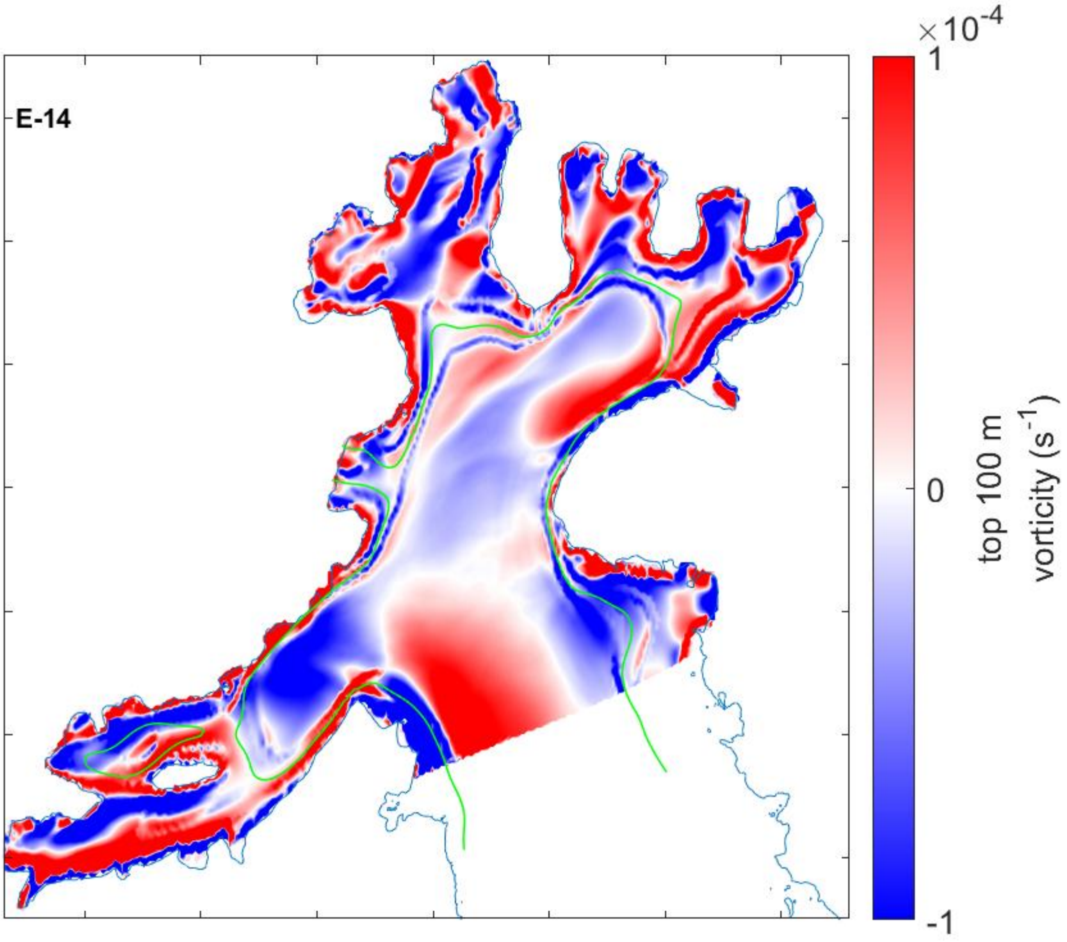

Figure S22. *As in Fig. S16, but for E-14 scenario.*

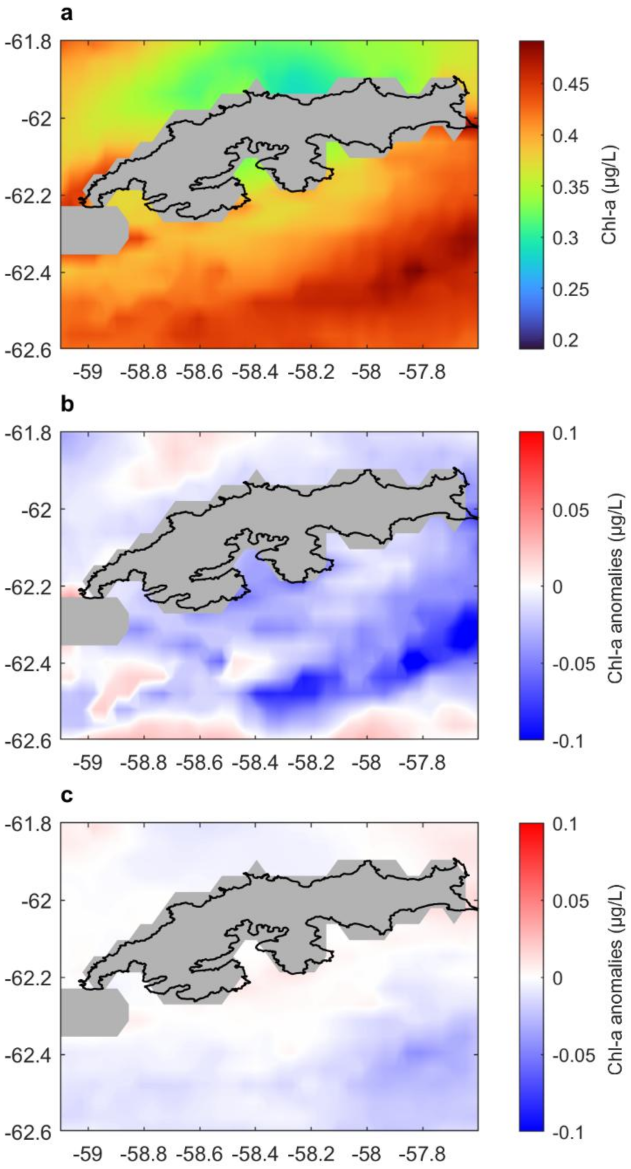

Figure S 23. *Chl-a estimates from the Copernicus-GlobColour dataset;* ***a.*** *mean values from December 15, 2018, to March 1, 2023;* ***b****. mean anomalies pertaining to* ***a.,*** *on days following days of predominant easterly wind impact;* ***c.*** *identical to b., but following days of westerly wind influence.*

Supplementary Video 1: *Video of a feeding hotspot of whales and penguins present at the mouth of Ezcurra Inlet on Jan 15th, 2022 (recorded by Maria Osińska)*
